# Conformational plasticity of SpyCas9 induced by AcrIIA4 and AcrIIA2: Insights from molecular dynamics simulation

**DOI:** 10.1016/j.csbj.2023.12.030

**Published:** 2023-12-27

**Authors:** Shuixiu Wen, Yuxin Zhao, Xinyu Qi, Mingzhu Cai, Kaisheng Huang, Hui Liu, De-Xin Kong

**Affiliations:** National Key Laboratory of Agricultural Microbiology, Agricultural Bioinformatics Key Laboratory of Hubei Province, College of Informatics, Huazhong Agricultural University, Wuhan, PR China

**Keywords:** CRISPR-Cas9, AcrIIA4, AcrIIA2, Conformational dynamics, Molecular dynamics simulations, SpyCas9

## Abstract

CRISPR-Cas9 systems constitute bacterial adaptive immune systems that protect against phage infections. Bacteriophages encode anti-CRISPR proteins (Acrs) that mitigate the bacterial immune response. However, the structural basis for their inhibitory actions from a molecular perspective remains elusive. In this study, through microsecond atomistic molecular dynamics simulations, we demonstrated the remarkable flexibility of *Streptococcus pyogenes* Cas9 (SpyCas9) and its conformational adaptability during interactions with AcrIIA4 and AcrIIA2. Specifically, we demonstrated that the binding of AcrIIA4 and AcrIIA2 to SpyCas9 induces a conformational rearrangement that causes spatial separation between the nuclease and cleavage sites, thus making the endonuclease inactive. This separation disrupts the transmission of signals between the protospacer adjacent motif recognition and nuclease domains, thereby impeding the efficient processing of double-stranded DNA. The simulation also reveals that AcrIIA4 and AcrIIA2 cause different structural variations of SpyCas9. Our research illuminates the precise mechanisms underlying the suppression of SpyCas9 by AcrIIA4 and AcrIIA2, thus presenting new possibilities for controlling genome editing with higher accuracy.

## Graphical abstract

**Figure:**
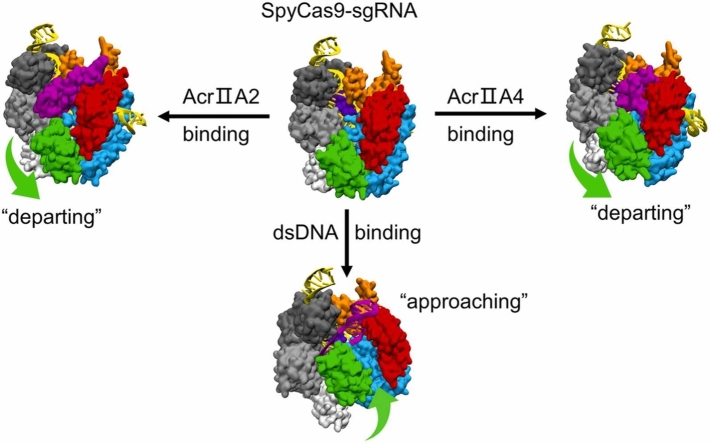

## 1 Introduction

CRISPR-Cas (clustered regularly interspaced short palindromic repeats and CRISPR-associated proteins) systems are adaptive immune systems in archaea and bacteria. Many exploit the dual RNA-guided DNA endonuclease Cas9 to defend against invading phages and conjugative plasmids [1], [2]. CRISPR-Cas immunity response involves three distinct mechanistic stages: adaptation, expression, and interference [3], [4], [5]. During the adaptation stage, also known as the spacer acquisition or immunization stage, a protospacer extracted from an invading foreign DNA is recognized through species-specific protospacer adjacent motifs (PAMs). Subsequently, this recognized protospacer is stored within the CRISPR array as a spacer. During the expression stage, or CRISPR RNAs (crRNAs) biogenesis stage, the CRISPR array is transcribed as precursor transcript (pre-crRNA), which undergoes endonucleolytic cleavage, yielding short mature crRNAs. The ultimate step is interference (antiviral defense or immunity stage), during which crRNAs guide a single or complex Cas nuclease for the specific cleavage of the complementary virus or plasmid target sequences that match the spacers. The CRISPR-Cas systems are partitioned into two classes: class 1 (including types I, III, and IV) with multisubunit effector complexes comprising several Cas proteins, and class 2 (including types II, V, and VI), in which the effector consists of a single, large, multidomain protein [6].

*Streptococcus pyogenes* Cas9 (SpyCas9), a single multidomain and multifunctional DNA endonuclease, belongs to type II CRISPR systems. It recruits double-stranded DNA (dsDNA) by utilizing the recognition of PAM and cleaves the DNA strands that are complementary and non-complementary to the 20-nt guide sequence in crRNAs (single guide RNA, sgRNA) with distinct nuclease domains HNH (His-Asn-His) and RuvC-like (RNase H fold), respectively [7], [8]. The overall structure of Cas9 encompasses a bilobed structure, encompassing an alpha-helical recognition lobe (REC) and a nuclease lobe (NUC) [8], [9], [10]. The REC lobe contains the REC1, REC2, and REC3 domains. In contrast, the NUC lobe contains a well-conserved domain HNH that adopts a one-metal-ion catalytic mechanism through its ββα-metal finger motif to cleave target strand DNA. This RuvC domain employs a two-metal ion mechanism to cleave non-target strand DNA, as well as a more variable C-terminal domain that displays a Cas9-specific fold and contains PAM-interacting (PI) sites required for PAM recognition and a wedge-like (WED) domain. The two lobes are connected through two linking segments, one formed by the arginine-rich region (BH, residues 59 to 76), which is crucial for sgRNA-DNA recognition, and the other disordered linker formed by residues 714 to 717 [9].

SpyCas9 harbors robust nuclease activity and target-specific DNA-complementary sgRNA, enabling precise genome engineering on a large scale in animal and cellular models [11], [12]. However, despite the radical advantages offered by the developing CRISPR-Cas toolbox, there are still several challenges to the efficacy and controllability of these technologies. These include the most prominent issue of off-target effects during in vivo or in vitro editing [13], cellular toxicity [14], and unexpected CRISPR on-target effects [15], all urgent problems to address to develop the accuracy of the technology. During the drastic coevolutionary arms race between CRISPR-Cas and Acrs, Acrs can interfere with CRISPR-Cas activities, providing temporal, spatial, and conditional control to neutralize and offset the excessive activity caused by the CRISPR-Cas effector as well as reducing the frequency of off-target effects, and can be delivered to cells to prevent unwelcome DNA cleavage due to the CRISPR-Cas system [16].

Anti-CRISPR proteins (Acrs), initially identified in 2013 [17], are natural CRISPR-Cas antagonists encoded by diverse mobile genetic elements (MGEs), such as plasmids and phages, inhibiting CRISPR-Cas immune function at various stages [18], [19], [20]. To date, over 88 anti-CRISPR proteins have been characterized within MGEs. The Acrs are classified based on the specific CRISPR-Cas system they inhibit [21], [22] and named according to their target subtype and the order of their discovery. For instance, AcrIIA4, a well-known anti-CRISPR protein, was the fourth protein identified to inhibit the type II-A CRISPR-Cas system. Biochemical and structural studies have revealed numerous strategies of anti-CRISPRs to counter CRISPR-Cas systems: preventing the acquisition of new CRISPR spacers, blocking the target DNA binding, inhibiting crRNA transcription or processing, inhibiting the CRISPR-Cas complex assembly, or preventing the target cleavage activity [21], [23]. Despite their small size (typically between 50 and 150 amino acids) no common features are conserved among these protein sequences.

**Fig. 1:**
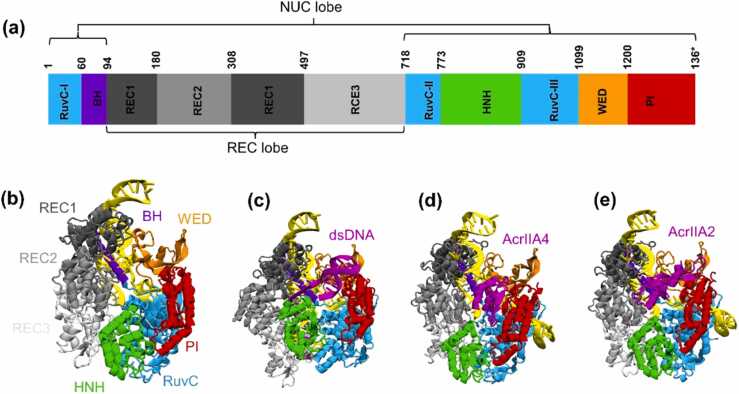
Overall structure of four different systems. (a) Schematic diagram of domain organization of SpyCas9. The architecture of the SpyCas9-sgRNA (b) (PDB ID: 4zt0, SpyCas9 with 1369 residues in total) in complex with dsDNA(c) (PDB ID: 4un3, SpyCas9 with 1372 residues in total), AcrIIA4 (d) (PDB ID: 5xbl, SpyCas9 with 1368 residues in total), and AcrIIA2 (e) (PDB ID: 6ifo, SpyCas9 with 1369 residues in total) shown in ribbon representation. SpyCas9 displays a bilobed structure encompassing an alpha-helical recognition lobe (REC) and a nuclease lobe (NUC). The REC lobe comprises the REC1, REC2, and REC3 domains, whereas the NUC lobe encompasses the RuvC homology (RuvC), bridge helix (BH), His-Asn-His (HNH), wedge-like (WED), and PAM-interacting (PI) domains.

Two of the most notable SpyCas9 inhibitors, AcrIIA2 and AcrIIA4, which belong to Class 2 anti-CRISPRs, successfully regulate gene-editing activities in human cells [24]. They restrain CRISPR-Cas immunity mainly by interfering with CRISPR-Cas complex assembly, inhibiting the binding of target DNA (or RNA), or preventing the cleavage of the target. The crystal structure of SpyCas9 in complex with a sgRNA and AcrIIA4 or AcrIIA2 is shown in Fig. 1. Structural and biochemical analysis revealed a mechanism similarity between AcrIIA2 and AcrIIA4: a DNA mimicry strategy helps them to repress the type II-A CRISPR-Cas system. AcrIIA4 occupies the PAM-interacting site in the PAM-interacting domain and shields the RuvC active site, blocking the recognition of dsDNA substrates by the Cas9-sgRNA complex [25]. Notably, AcrIIA2 is functionally equivalent to AcrIIA4, occupying the PAM-binding pocket but also locking the HNH domain, preventing it from rotating toward the REC lobe upon target DNA binding, disabling the function of the CRISPR-Cas system [26]. Altogether, structural and biochemical studies have offered vast amounts of detail about the inhibition mechanisms of AcrIIA2 and AcrIIA4, the most widely used genome-editing tool, opening new horizons of regulatory precision during genome editing. However, it is unknown how structural plasticity could contribute to the rearrangement of the SpyCas9-sgRNA binary complex when AcrIIA2 and AcrIIA4 are expressed in bacterial cells. Moreover, it remains unclear why the sgRNA-bound SpyCas9, but not the free one, is inhibited by AcrIIA2 and AcrIIA4.

Here, through an extensive multi-microsecond molecular dynamics (MD) simulation of the CRISPR-Cas9 complex with AcrIIA2 and AcrIIA4, we sought to gain a comprehensive insight into the conformational plasticity of the protein complexes and to elucidate the dynamic relationship between them over extended timescales. The results indicate that the interaction of AcrIIA4 and AcrIIA2 with SpyCas9 endonuclease causes a conformational shift towards an inactive “departing” state, leading to spatial separation between the nuclease and cleavage sites and hindering the propagation of signals in the PAM recognition and nuclease domains. Through these detailed investigations, we provide valuable insights into the precise molecular mechanisms underlying the inhibition of SpyCas9 by AcrIIA4 and AcrIIA2, offering novel prospects for fine-tuning genome-editing processes with enhanced precision.

## 2 Materials and methods

### 2.1 Structural Models

MD simulations have been performed on four model systems of CRISPR-Cas9 based on the available crystal structures (Fig. 1). The RNA-bound state has been resolved from SpyCas9 in complex with an 85-nt sgRNA at a resolution of 2.9 Å (PDB ID: 4zt0) [27]. The DNA-bound form is based on the X-ray structure of the SpyCas9-sgRNA-dsDNA complex solved at a resolution of 2.59 Å (PDB ID: 4un3) [28]. The Acr-bound models are prepared based on two X-ray structures: the SpyCas9-sgRNA-AcrIIA4 complex determined at a resolution of 3.0 Å (PDB ID: 5xbl) [25] and the SpyCas9-sgRNA-AcrIIA2 complex solved at a resolution of 3.3 Å (PDB ID: 6ifo) [26]. The missing residues of the X-ray structures were completed via comparative modeling using the original structure (without the missing residues) as a template via the MODELLER program (v9.25), and the crystallization waters were retained in the final models. The composition of residues or nucleotides for the four models is provided in Table S1. The solvated systems containing SpyCas9-sgRNA, SpyCas9-sgRNA-dsDNA, SpyCas9-sgRNA-AcrIIA4, and SpyCas9-sgRNA-AcrIIA2 exhibited sizes of approximately 163,000, 150,000, 153,000, and 158,000 atoms, respectively.

### 2.2 Molecular dynamics simulation protocols

The model systems were simulated using conventional MD simulation, employing the AMBER20 software. The amber force fields ff14SB [29] and OL3 [30] were chosen to describe the ribonucleoprotein (RNP) complex and RNA, respectively. All systems were immersed in a TIP3P water box, with a minimum thickness of 12.0 Å from the boundaries. For all systems, Na^+^ and Cl^−^ were added to neutralize the system while imitating the in vivo physiological cleavage condition. The topology and coordinate files were created using TLEAP. Periodic boundary conditions were implemented in the simulations, and the particle mesh Ewald method was employed to calculate long-range electrostatic interactions accurately. A cutoff of 9 Å was implemented to incorporate van der Waals interactions. Before the production runs, the systems first underwent energy minimization using the steepest descent algorithm. Subsequently, all systems were equilibrated in a canonical ensemble (NVT) for 600 ps after heating from 0 K to 300 K within 50 ps. The production runs involved each system reaching 1 μs, resulting in 12 μs of classical MD (1 μs × 3 replicas × 4 systems).

### 2.3 Principal component analysis

Principal component analysis (PCA) is a method that can describe large-scale collective motions occurring in biological macromolecules within the context of MD simulations. In PCA, the covariance matrix of the protein Cα atoms is calculated and diagonalized to obtain a reduced set of generalized coordinates (eigenvectors) to describe the system’s motions. Eigenvectors, also known as principal components (PCs), represent the mean square fluctuation of the system when its trajectory is projected onto that specific eigenvector. Ordering the eigenvectors by their eigenvalues enables the identification of the first principal component (PC1) as the dominant motion with the largest amplitude, often referred to as the essential dynamics of the system [31]. In our analysis, the MD simulation trajectories of the four systems (SpyCas9-sgRNA, SpyCas9-sgRNA-dsDNA, SpyCas9-sgRNA-AcrIIA4, and SpyCas9-sgRNA-AcrIIA2) stripped down to Cα carbons only were superposed onto the same reference structure as previously described [32], and then projected into the collective coordinate space defined by the first two eigenvectors (PC1 and PC2), enabling us to characterize the essential conformational subspace sampled by Cas9 during MD and identify the distinctions of the essential dynamic properties of Cas9. PCA was conducted using the Cpptraj program in Amber20, and the Normal Mode Wizard (NMWiz) plugin in VMD was employed to analyze the normal mode data derived from the essential dynamics analysis of the MD trajectories.

### 2.4 Cross-correlation analysis

The evaluation of the cross-correlation (CC_ij_) between Cα atoms (i and j) is an informative approach that reveals the dynamical coupling correlation of the atomic fluctuations and provides a specific method for comparing diverse simulations by considering the correlation and magnitude of the atomic motions, that is, the diagonal elements of the covariance matrix. The CC_ij_ analysis is based on Pearson coefficients, calculated by obtaining the ensemble average of the position vectors ${∆r}_{i}$ and ${∆r}_{j}$ for Cα atoms i and j, respectively. The covariance c_ij_ is determined using the following formula [33]:

(1) $$c_{\mathrm{ij}}=<{∆r}_{i}.{∆r}_{j}>$$

The cross-correlation, or normalized covariance, is given by the formula:

(2) $${\mathrm{CC}}_{\mathrm{ij}}=\frac{{\mathrm{cc}}_{\mathrm{ij}}}{{\left[{\mathrm{cc}}_{\mathrm{ii}}.{\mathrm{cc}}_{\mathrm{jj}}\right]}^{1/2}}=\frac{<{∆r}_{i}.{∆r}_{j}>}{{<{{∆r}_{i}}^{2}>}^{1/2}.{<{{∆r}_{j}}^{2}>}^{1/2}\;}$$

The degree of correlation between atomic motions, as indicated by the Cross-Correlation (CC_ij_) of Cα atomic fluctuations between residue pairs (i and j), provides crucial insights into the dynamic coupling and comparative analysis of multiple simulations. Specifically, the CC_ij_ values reflect the phase and period similarity of the atomic motions: CC_ij_ = 1 or − 1 indicate complete correlation or anti-correlation, respectively, while CC_ij_ = 0 suggests motions that are out of phase (that is, the positions are oriented at an angle of 90°) but have the same period. Positive CC_ij_ values signify lockstep motions between the residues, whereas negative CC_ij_ values describe anticorrelated motions. To investigate the correlated motions of each residue, the CC_ij_ matrices were computed for each system from ∼1 μs trajectories independently and then extended to three independent MD replicas. The resulting CC_ij_ coefficients were utilized to construct matrices that visually represent the correlated motions of each residue with one another.

### 2.5 Dynamic network analysis

Community network analysis (CAN) of correlated protein motions offers a powerful tool for modeling signal propagation in protein systems [32], [34], [35]. To construct a protein graph for CAN, each protein residue is represented as a “node,” and edges are formed between nodes corresponding to residues in contact with one another based on the correlation coefficients computed from the Cα atomic position fluctuations. The weight of each edge is determined by the residue-pair correlation [36], defined as

(3) $$d_{\mathrm{ij}}=-\log|{\mathrm{CC}}_{\mathrm{ij}}|$$

where d_ij_ represents the “distance” between nodes i and j, and CC_ij_ is their pairwise correlation. The Girvan–Newman algorithm is then used to construct the community structure of each graph. For two nodes to be considered connected, at least one heavy atom of the two residues must be within a cutoff distance of 5 Å for at least 75% of the simulation time, which consists of all equilibrated simulations (the last 1 μs in 3 replicas, 12 μs in total) for each system. Dijkstra’s algorithm in NetworkX can calculate the shortest pathways (SPs) within the protein communication network, representing the most efficient communication pathways between pairs of residues. Finally, the “modularity” parameter Q, which measures the difference in probability between intra- and inter-community edges, is used as a metric for assessing the quality of the community structure. Higher Q values correspond to more distinct community structures, with a maximum value of 1.

## 3 Results and discussion

### 3.1 Conformational plasticity of SpyCas9 combined with AcrIIA4 and AcrIIA2

To study the inhibitory mechanism of SpyCas9 by anti-CRISPR AcrIIA4 and AcrIIA2 at the atomic level, microsecond-length MD simulations were performed on the available X-ray structures, including SpyCas9 in complex with RNA (SpyCas9-sgRNA), with dsDNA (SpyCas9-sgRNA-dsDNA), and with Acr proteins (SpyCas9-sgRNA-AcrIIA4 and SpyCas9-sgRNA-AcrIIA2). For the systems of SpyCas9, MD simulations were executed in explicit solvent, obtaining multiple μs-length trajectories (that is, three replicas of 1 μs each) with an overall sampling of ∼12 μs.

Root-mean-square fluctuation (RMSF) and root-mean-square deviation (RMSD) analyses indicate that upon AcrIIA4 and AcrIIA2 binding, the overall conformational flexibility of the protein is preserved, but the flexibility of different protein domains changes intricately. RMSF analysis, a traditional method of measuring protein flexibility, was used to understand the overall flexibility of the system combined with AcrIIA4 and AcrIIA2 (Fig. S1). Despite all the significant residue fluctuations of SpyCas9 domains in the presence of AcrIIA4 and AcrIIA2, the most conspicuous undulation of RMSF was observed in the RuvC-III and WED domains of all systems. Moreover, compared to the other three systems, the overall vibration of domains in SpyCas9-sgRNA-AcrIIA2 was reduced. The results of RMSF show that the SpyCas9-sgRNA-AcrIIA2 system was more stable during the simulation. The binding of Acr proteins weakens the flexibility of each domain of the Cas protein. RMSD values with respect to the SpyCas9′s Cα atoms of the four systems are displayed in Fig. S2. In all replicas of the four systems, the RMSD of Cα atoms fluctuates within the range of 3–5 Å.

### 3.2 Conformational transition and ensemble

**Fig. 2:**
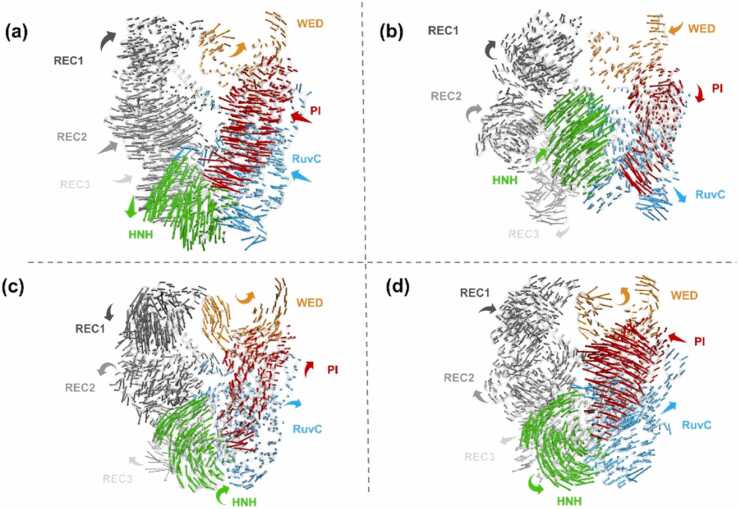
Essential dynamics described along the first principal component (PC1) of the individual protein domains of the SpyCas9-sgRNA (a), SpyCas9-sgRNA-dsDNA (b), SpyCas9-sgRNA-AcrIIA4 (c), and SpyCas9-sgRNA-AcrIIA2(d) systems, shown using arrows to indicate the relative direction and amplitude of the motions.

PCA was performed to dissect the large-scale collective motions of the SpyCas9 domains in different states and depict the global conformational transition of SpyCas9. The essential dynamics of SpyCas9 along the first principal mode of motion (i.e., PC1) are shown in Fig. 2, where the arrows indicate the relative direction and amplitude of the motions. Compared with SpyCas9-sgRNA, large amplitude motions of SpyCas9 domains were observed directly in the association process with AcrIIA4 and AcrIIA2: the position and amplitude of almost all domains of SpyCas9 were different when associating with AcrIIA4 or AcrIIA2 compared to SpyCas9 binding with sgRNA only, indicating that configuration rearrangement occurred in almost all domains of SpyCas9 to varying degrees upon binding with AcrIIA4 and AcrIIA2 (Fig. 2c, d). Furthermore, the discrepancy of amplitude and direction of domains in the systems of SpyCas9-sgRNA-AcrIIA4 and SpyCas9-sgRNA-AcrIIA2 also existed. The amplitude of the HNH domain in the SpyCas9-sgRNA-AcrIIA2 system is much larger than in the SpyCas9-sgRNA-AcrIIA4 system, but for the REC2 domain, the reverse occurs, indicating a distinct domain bias for the structural changes of SpyCas9 induced by AcrIIA2 and AcrIIA4. Interestingly, the catalytic RuvC domain exhibits small amplitude motions in all systems, indicating a high structural stability of the conserved protein domain [37].

Further insights into the domain transformation of SpyCas9 binding with dsDNA along PC1 revealed an “approaching” conformation, which is characterized mainly by the systematic relocation of the HNH toward the cleavage state (Fig. 2b and Supplementary Movie S2). In addition, the “departing” conformations of AcrIIA4- and AcrIIA2-bound complexes were defined mainly by the unsuccessful relocation of the HNH toward the cleavage state (Fig. 2c, d, and Supplementary Movies S3, S4). Learning from the “approaching” conformation of the SpyCas9-sgRNA-dsDNA system, we found that the HNH, REC2, and PI domains synchronously move towards the dsDNA at the hollow of SpyCas9, in agreement with the structural rearrangements required for PAM recognition and nucleic acid target cleavage [38]. Previous experimental studies revealed that the HNH domain transitions between multiple conformations before recognizing its on-target DNA, which can help HNH accurately dock into its active state [39], [40]. In the departing conformation of AcrIIA4- and AcrIIA2-bound systems, the HNH domain departed away from the central concave region responsible for RNA/DNA heteroduplex accommodation to varying degrees, elongating the spatiality between the REC, HNH, and RuvC domains; this is a typical state of catalytically inactive SpyCas9 [38]. Similarly, the departing conformation occurs in AcrIIA6 binding with St1Cas9-sgRNA [34].

**Fig. 3:**
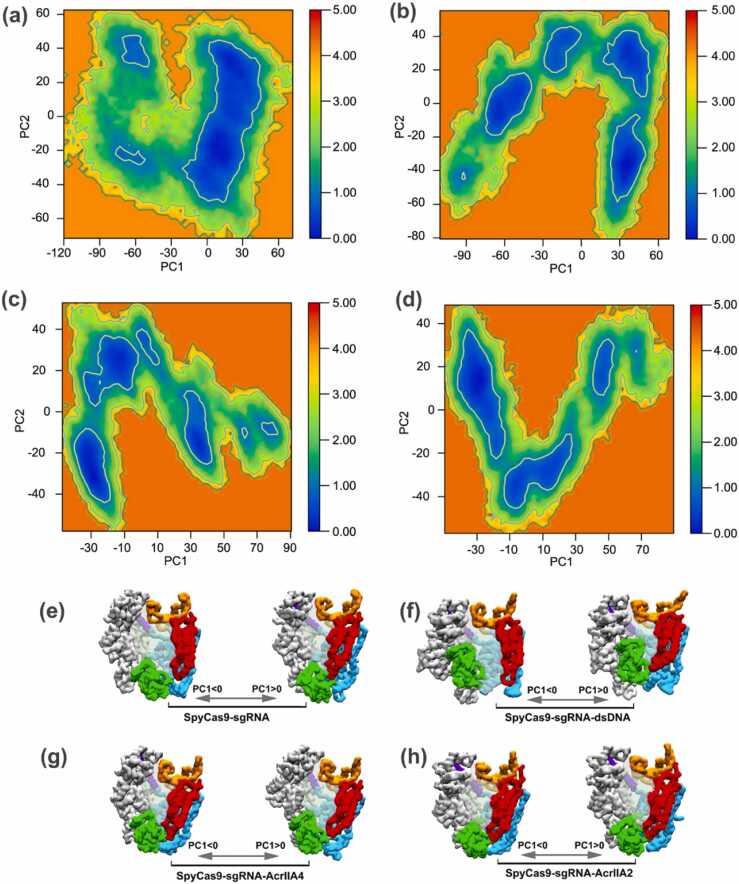
The free energy landscape of SpyCas9-sgRNA (a), SpyCas9-sgRNA-dsDNA (b), SpyCas9-sgRNA-AcrIIA4 (c), and SpyCas9-sgRNA-AcrIIA2 (d) systems were plotted using the first two principal components. (e-h) Representation of the conformation structure of each complex along PC1. Structures were aligned and rendered with the same viewing angle.

The free energy landscape (FEL) plotted by the first two principal components (PC1 and PC2) was utilized to explore the conformational ensembles near the native state structure. Fig. 3a–d display the FELs of SpyCas9 bond to sgRNA only, dsDNA, AcrIIA4, and AcrIIA2, where the deeper blue indicates the more stable conformational ensembles having lower energy. The conformational spaces with significant differences, identified as those closest to and farthest from the original conformation of SpyCas9 in each system, were primarily determined utilizing PC1 (Fig. 3e-h). We observed that SpyCas9-sgRNA only has a single global minimum confined within a local basin (Fig. 3a). The free-energy profiles of AcrIIA4- and AcrIIA2-bound SpyCas9 systems in the two two-dimensional projections do not trigger multiple minima and are very similar, featuring three main energy basins differing by about 1 kJ/mol, indicating different conformational motions (Fig. 3c, d). Analysis of the conformational ensembles derived from FELs of the four complexes revealed that AcrIIA4- and AcrIIA2-bound SpyCas9 were more stable compared to sgRNA-bound SpyCas9, suggesting that AcrIIA4 and AcrIIA2 binding does not induce significant conformational changes in the sgRNA-bound SpyCas9, in agreement with previous experimental studies [25], [26]. Additionally, since the radius of gyration is a characteristic of the compactness of protein structures, a similar result can be observed from the radius of gyration calculated considering the protein Cα atoms of all four systems (Fig. S4).

Overall, the results suggest that SpyCas9 undergoes a “departing” conformational transition associated with AcrIIA4 and AcrIIA2 but an “approaching” conformational transition while binding with dsDNA. Most strikingly, in addition to the conspicuous configuration rearrangement of the PI and REC2 domains, AcrIIA4 and AcrIIA2 triggered the departure of the HNH domain away from the central concave region responsible for RNA/DNA heteroduplex accommodation with varying degrees of magnitude, increasing the difficulty of on-target DNA identification and ultimately decreasing the catalytic activity of SpyCas9.

### 3.3 Correlated motions between SpyCas9 domains mediate inhibition mechanism of AcrIIA4 and AcrIIA2

**Fig. 4:**
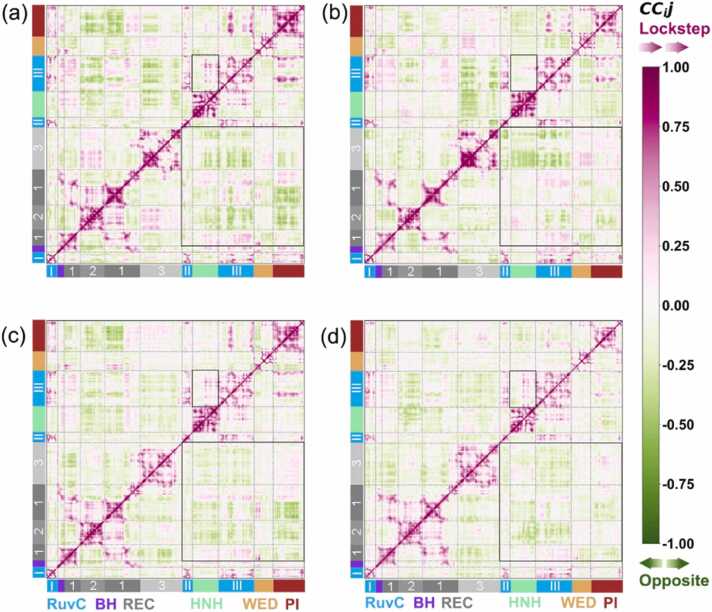
Correlated motions of SpyCas9-sgRNA (a), SpyCas9-sgRNA-dsDNA(b), SpyCas9-sgRNA-AcrIIA4 (c), and SpyCas9-sgRNA-AcrIIA2 (d) systems. The data were averaged over three simulation replicas, each lasting approximately 1 μs. The results of each replica can be observed in Fig. S6. The magnitude of the CC_ij_ is colored from magenta (for CC_ij_ ≥ 0, indicating lockstep motions) to green (for CC_ij_ ≤ 0, indicating opposite motions). Black boxes highlight anticorrelated CC_ij_ motions between the REC and NUC lobes.

Pearson cross-correlation coefficient (CC_ij_) analysis was used to investigate the possible dynamic correlations between different domains of SpyCas9. The results indicated that the binding of substrate DNA, AcrIIA4, and AcrIIA2 reshaped the overall correlation between the protein domains (Fig. 4). The jumble of correlated and anticorrelated motions between the REC and NUC lobes (including RuvC, HNH, PI, and WED) indicated the tendency of the REC lobe to move in lockstep (or the opposite) way with respect to the NUC lobe (highlighted by black boxes in the lower triangular), again supporting the departing conformational transition underlying Acr protein binding. Similar results were reported in previous studies [32], [41], [42].

The nuclease activity of the HNH and RuvC domains is coupled, that is, HNH conformational changes trigger RuvC domain nuclease activity, indicating a tight dynamic “cross-talk” between the HNH and RuvC domains [40], [43]. Here, compared with sgRNA-bound pre-targeting (Fig. 4a) and substrate DNA binding checkpoint states (Fig. 4b), the association with AcrIIA4 and AcrIIA2 proteins partly induced the enhancement of correlated movements between the HNH and RuvC domains of SpyCas9 (highlighted by black boxes in the upper triangle), which may imply that the “cross-talk” activity also occurs in anti-CRISPR binding systems. Previous studies have revealed strong correlations between the HNH and REC domains, indicating that the conformational changes of the REC lobe facilitate the docking of the HNH domain at the target-strand DNA [44]. The weakness in part due to the correlation between HNH and REC domains in our study unveiled that the association of AcrIIA4 and AcrIIA2 may disturb the conformational transformation of the REC lobe and then hamper the conformational transitions of the HNH domain toward the cleavage state. In addition, compared to sgRNA-bound only, the association with DNA and the anti-CRISPRs significantly enhanced correlations between the PI and REC3 domains of SpyCas9; however, a smaller increase was observed in the AcrIIA2-binding system. It also appears that the inter-correlation of domains of SpyCas9 binding with AcrIIA2 is different from that of AcrIIA4 binding.

Collectively, the weakest inter-domain correlation was observed in SpyCas9-sgRNA. Meanwhile, the NUC and REC lobes established a stronger correlation upon substrate DNA, AcrIIA4, and AcrIIA2 loading, indicating the key role of REC in coordinating nuclease activity in the Acr-induced inhibition mechanism. Otherwise, the enhanced correlation between the HNH and REC domains implies that the “cross-talk” activity also occurs in AcrIIA4- and AcrIIA2-binding systems. The coupled motions and the essential dynamics of the individual SpyCas9 domains indicate their tendency to move concertedly in different directions to allow the conformational transition accommodating the binding of substrate DNA or Acr proteins.

### 3.4 Determination of crucial residues involved in AcrIIA4 and AcrIIA2 protein binding

**Fig. 5:**
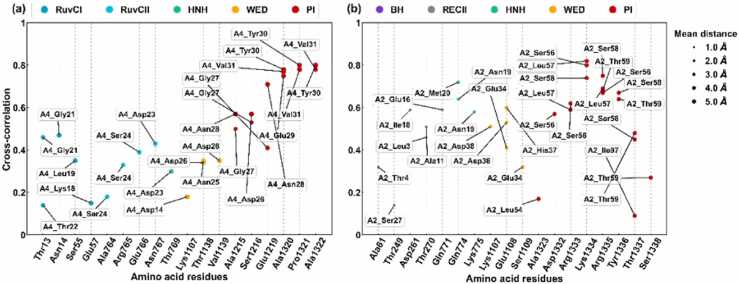
Cross-correlation coefficients for the frequent contacts between anti-CRISPR (AcrIIA4 (a) and AcrIIA2 (b)) and amino acid residues in the SpyCas9 protein. Two residues are considered to be frequently contacted if any of their heavy atoms are within 8 Å of each other for at least 75% of the simulation time. The x-axis represents the amino acid residue of SpyCas9, and the y-axis indicates the cross-correlation coefficient. Labels indicate the frequent contact residues of anti-CRISPRs, the circle size indicates the average Cartesian distance of Cα between SpyCas9 and the anti-CRISPRs, and colors discriminate the domain of residue of SpyCas9.

Previous experimental studies revealed that AcrIIA2 and AcrIIA4 function similarly, that is, they repress the type II-A CRISPR-Cas system via DNA mimicry [25], [26]. However, minor differences exist in the structural changes of SpyCas9 induced by AcrIIA2 and AcrIIA4, as revealed by detecting amino acid interactions. In Fig. 5, we show all contacted residues with cross-correlation coefficients that cover key residues of SpyCas9 involved in Acr recognition. Here, a pair of residues is in contact if any heavy atom of the pair of residues is within 8 Å for at least 75% of the simulation time.

Fig. 5 and S7 show that many of the crucial residues in SpyCas9 have a high correlation of contact with residues in AcrIIA4 and AcrIIA2 and exhibit interactions made by the same amino acids. For sgRNA-bound SpyCas9 by AcrIIA4 (Fig. 5a, S7a, and S7b), residues Gly21, Asp23, and Ser24 in the β1-β2 loop of the bound AcrIIA4 have a high correlation contact with residues Thr13, Asn14 in the RuvC-I domain, and Ala764, Arg765, Glu766, Asn767, Gln768, and Thr769 in the RuvC-II domain. In addition, residues Tyr30, Val31 in the β2 strand and Asn40, Glu41, Tyr42, and Val43 in the β3 strand of the bound AcrIIA4 have a high correlation interaction with Ala1320, Pro1321, and Ala1322 in the PI domain. For sgRNA-bound SpyCas9 by AcrIIA2 (Fig. 5b, S7c, and S7d), residues Glu34, His37, and Asp38 of the bound AcrIIA2 have a high correlation contact with the residues Lys1107, Glu1108, and Ser1109 in the WED domain. Moreover, residues Ser56, Leu57, Ser58, and Thr59 of the bound AcrIIA2 have a high correlation interaction with residues Asp1332, Arg1333, Lys1334, Arg1335, Tyr1336, and Thr1337 in the PI domain.

Through simulation, distinct binding traits of several key residues verified experimentally were reaffirmed, for example, AcrIIA2 interacts with residues crucial for PAM recognition (Arg1333 and Arg1335 of SpyCas9); however, regrettably, we did not capture the contacts between AcrIIA4 and the PAM recognition residues. To accurately identify the available residues (hotspots) in both the two anti-CRISPRs and SpyCas9, we conducted a hydrogen bond analysis. In this analysis, geometric considerations (a distance cutoff radius of 3.5 Å and an angle cutoff of 135°) were used to determine whether a hydrogen bond was formed. Hydrogen bond interactions were considered stable and pervasive if there was a hydrogen bond between all possible donors and acceptors for at least 30% of the simulation time and in two of the three repetitions.

**Fig. 6:**
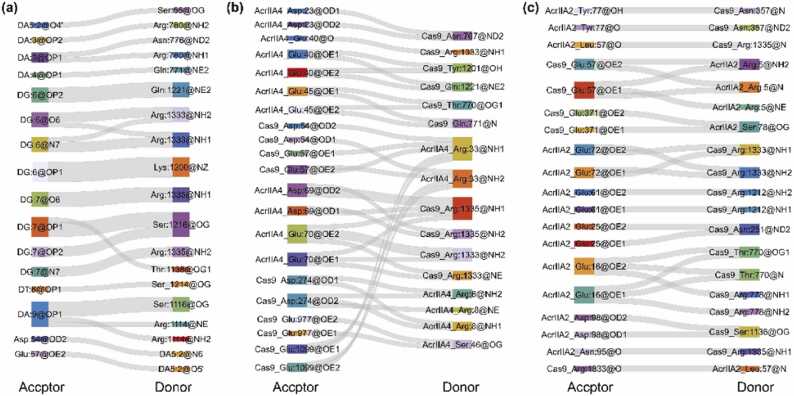
Hydrogen bond interactions of sgRNA-bound SpyCas9 by dsDNA (a), AcrIIA4 (b), and AcrIIA2 (c). Geometric considerations, i.e., a distance cutoff radius of 3.5 Å and an angle cutoff of 135°, were used to determine whether a hydrogen bond was formed. Hydrogen bond interactions were considered stable and pervasive if there was a hydrogen bond between all possible donors and acceptors for at least 30% of the simulation time and in two of the three repetitions.

Hydrogen bonds can be distinguished as strong (2.2–2.5 Å), moderate (2.5–3.2 Å), and weak strengths (3.2–4.0 Å) [45]. Most hydrogen bonds between SpyCas9 and dsDNA, AcrIIA4, and AcrIIA2 are moderately strong (Fig. S8). Fig. 6a shows that there are several hydrogen bonds formed between the guanine nucleobases of DG6 and DG7 in the non-target strand and Lys1200, Gln1221, Arg1333, Arg1335, Ser1216, and Thr1138, reaffirming the biochemical experiment results that the non-complementary strand GG PAM dinucleotides interact with the conserved arginine residues of SpyCas9 [28]. Residues Arg8, Asp23, Arg33, Glu45, and Ser46 of the bound AcrIIA4 are involved in a hydrogen-bonding network with residues Asp274, Asn766, Asp52, Glu57, Glu1099, Thr770, Gln771, and Glu977 of the RuvC domain of SpyCas9 (Fig. 6b). Notably, residues Arg1333 and Arg1335 in the PI domain, which are vital for recognizing the PAM sequence in the SpyCas9-sgRNA-dsDNA ternary complex [28], form a hydrogen-bonding network with residues Asp69 and Glu70 in the α2–α3 loop of the bound AcrIIA4 instead (Fig. 6b). Hydrogen bonds are also populated between residue Glu40 of the bound AcrIIA4 and residues Tyr1201, Gln1221, and Arg1333 in the PI domain. These results agree with the crystallographic studies of AcrIIA4 bound to SpyCas9-sgRNA binary complexes [25], [46]. Extensive hydrogen bonds also formed between SpyCas9 and AcrIIA2 (Fig. 6c). Similar to AcrIIA4, the PI domain of SpyCas9 forms a comprehensive hydrogen interaction network with AcrIIA2. In the AcrIIA2-bound SpyCas9-sgRNA complex, residues Arg1212, Arg1335, and Arg1333 form stabilized hydrogen bonds with the residues Glu61, Leu57, and Glu72 of AcrIIA2, respectively. Following this, there were stable hydrogen bonds between residues Tyr77, Ser78, and Glu25 of AcrIIA2 and the residues Asn357, Glu371, and Asn251 of the REC domain of SpyCas9. More importantly, a hydrogen bond between residue Glu16 of AcrIIA2 and residue Thr770 of the HNH domain helps to distinguish the binding-change mechanism of AcrIIA2 and AcrIIA4 responsible for inhibiting SpyCas9 function.

**Fig. 7:**
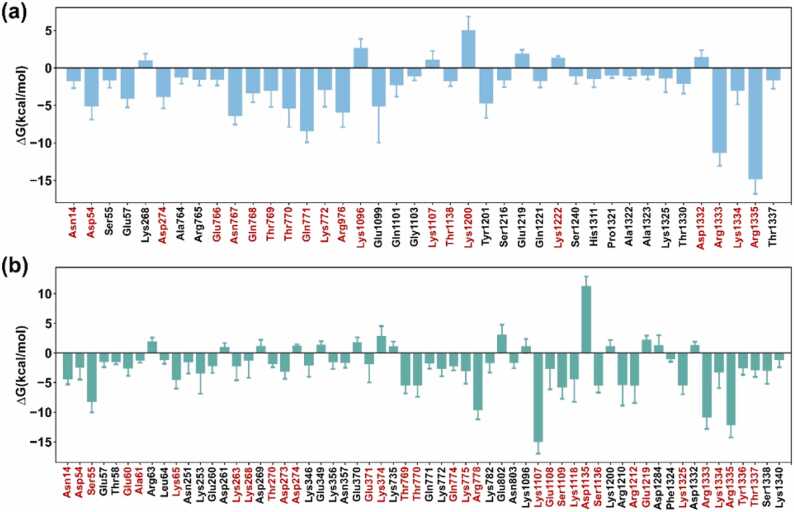
Binding energy contributions of residues in SpyCas9 to AcrIIA4 (a) and AcrIIA2 (b). Residues with prominent binding energy contribution in three replicas (> 1 kcal/mol or < −1 kcal/mol) are selected. If the same residue occurred in at least two replicates, the energy contribution with the highest rate was chosen as the ultimate outcome, and the corresponding residue was annotated in red.

The frames extracted from the last 100 ns trajectories were applied to the MM/GBSA binding free energy calculation for AcrIIA4- and AcrIIA2-bound SpyCas9 systems. The predicted ∆G of SpyCas9 to AcrIIA4 was − 80.54 ± 0.29 kcal/mol, and that of AcrIIA2 is − 74.20 ± 0.34 kcal/mol, suggesting that AcrIIA2 binds sgRNA-loaded SpyCas9 with lower affinity than AcrIIA4, aligning well with the experiment result [47]. To elucidate the residues contributing to changes in the overall binding affinity, we conducted a per-residue binding energy decomposition analysis (Fig. S14). In Fig. 7, residues with prominent binding energy contribution in three replicas (> 1 kcal/mol or < −1 kcal/mol) were selected. If a residue appeared in at least two replicates, the highest energy contribution was selected as the final outcome, and the corresponding residue was annotated in red. For AcrIIA4-bound SpyCas9, 15 key residues with energy contributions < −1.00 kcal/mol were identified, including Asn14 and Asp54 in the RuvC-I domain, Glu766, Asn767, Gln768, Thr769, Thr770, Gln771, and Lys772 in the RuvC-II domain, and Arg1333, Lys1334, and Arg1335 in the PI domain. Similarly, more than 20 pivotal residues were found in SpyCas9 with binding affinity < −1.00 kcal/mol to AcrIIA2, including Asn14, Asp54, Ser55, and Glu60 of RuvC-I domain; Lys65, Lys263, Lys268, Thr270, Asp273, and Glu371 of REC domain; Thr769 and Thr770 of RuvC-II domain; Gln774, Lys775, and Arg778 of HNH domain; Lys1107, Glu1108, Ser1109, Lys1118, and Ser1136 of WED domain; Arg1212, Glu1219, Lys1325, Agr1333, Lys1334, Arg1335, Tyr1336, and Thr1337 of PI domain. Moreover, residues Asn14, Asp54, Thr769, Thr770, Gln771, Lys772, Arg1333, Lys1334, and Arg1335 were identified as crucial for binding affinity in both AcrIIA4- and AcrIIA2-bound SpyCas9 systems. To summarize, residue contacts with cross-correlation, further hydrogen analysis, and per-residue binding energy decomposition analysis present the crucial residues in the process ofAcrIIA4 and AcrIIA2 binding to sgRNA-bound SpyCas9. Arg1333 and Arg1335 of SpyCas9 are key residues for PAM recognition; both AcrIIA2 and AcrIIA4 interact with these two residues with high cross-correlation coefficients and extensive hydrogen bonds blocking the recognition of SpyCas9 and the PAM sequence. Furthermore, the aforementioned analysis identified crucial residues of SpyCas9 interacting with AcrIIA4, including Asn14, Asp54, Thr770, and Gln771, previously overlooked in the crystal structure [25]. Similarly, the examination of SpyCas9 interacting with AcrIIA2 identified key residues, including Asn14, Asp54, Ser55, Lys775, Arg1212, and Glu1219 [26]. Differences in the binding-change mechanism of SpyCas9 induced by AcrIIA4 and AcrIIA2 were also easily visible. In addition to forming hydrogen bonds with the PI and REC domains, AcrIIA2 interacts with the HNH domain, but AcrIIA4 interacts with the RuvC domain.

### 3.5 AcrIIA4 and AcrIIA2-mediated information flow in SpyCas9

Community network analysis provides valuable insights into correlated protein motions, allowing for the reorganization of conformations and the propagation of the signal in protein systems [32], [34], [35]. The dynamical weighted network, described by a set of nodes (Cα of each amino acid residue) and edges weighted according to CC_ij_, can be split into “communities” by applying the Girvan–Newman algorithm, local substructures involving groups of nodes within which the connections are strong and dense but between which are relatively weaker and sparser. The acquired community networks were represented as interconnected spheres linked by sticks, where the thickness was proportional to the number of shortest paths traversing those junctions.

**Fig. 8:**
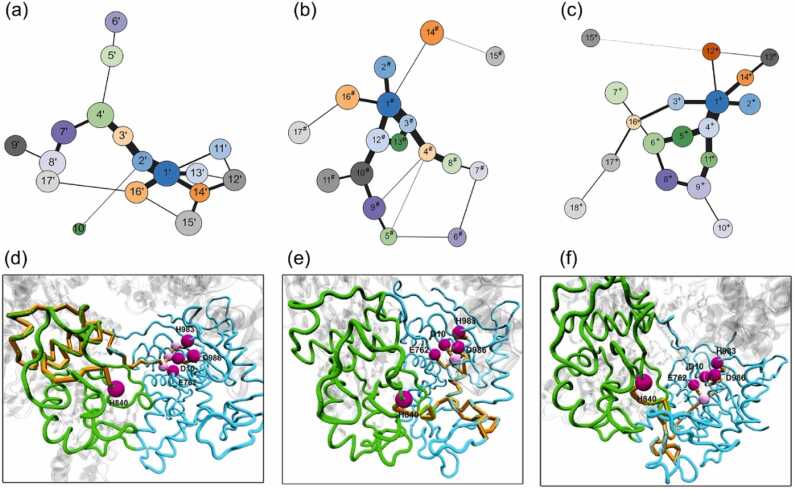
Community network representation of SpyCas9-sgRNA-dsDNA (a), SpyCas9-sgRNA-AcrIIA4 (b), and SpyCas9-sgRNA-AcrIIA2 (c) systems. The acquired community networks were represented as interconnected spheres linked by sticks, where the thickness was proportional to the number of shortest paths traversing those junctions. “Shortest pathways” linking catalytic residues in the RuvC and HNH domains of three Cas9 systems: SpyCas9-sgRNA-dsDNA (d), SpyCas9-sgRNA-AcrIIA4 (e), and SpyCas9-sgRNA-AcrIIA2 (f). The catalytic residues of the RuvC domain (E762, D986, D10, H983) were designated as the “source,” and the catalytic residue of the HNH domain (H840) as the “sink” to trace the flow of information from the sources to the sink. The 3D structures of Cas9 were used to depict the pathways using lines, and the residues forming the pathways (Cα atoms) were shown using spheres. The characters “',” “#,” and “+ ” are used to distinguish community compositions in different systems.

The Girvan–Newman algorithm splits the network of SpyCas9-sgRNA, SpyCas9-sgRNA-dsDNA, SpyCas9-sgRNA-AcrIIA4, and SpyCas9-sgRNA-AcrIIA2 into 19, 17, 17, and 18 communities, respectively (Fig. 8 and Table S2). In the RNA-bound states, community 9, which is related to the BH domain, functions as a significant hub that facilitates the flow of information between the REC and NUC lobes (Figure. S9a). Additionally, community 1, which represents the RuvC domain, plays a central role in the information flow by linking the HNH and PI domains. The appearance of dsDNA, AcrIIA4, and AcrIIA2 reduces the number of communities in SpyCas9 and enhances the flow of signaling (Fig. 8a–c). With loading dsDNA, the PI domain-containing community 16 showed a stronger correlation with the RuvC domain (community 1). Moreover, the HNH and RuvC domains were identified as highly interconnected communities consistent with the experimentally observed tight correlation in catalytic activity between them, providing compelling evidence for communication [40].

With loading AcrIIA4, the transfer of stronger information occurred between the PI and RuvC domains, as evidenced by the established stronger correlation between community 1, which represents the RuvC domain alone, and community 2, which contains residues from both the PI and RuvC domains. Furthermore, unlike dsDNA loading, an enhanced information flow is observed between the RuvC-III and REC3 domains, indicated by the thick stick connecting communities 12 and 10, representing the RuvC-III and the REC3 domain, respectively. A similar tendency was observed during AcrIIA2 loading, with a stronger information transaction occurring between the PI and RuvC domains and RuvC-II and REC3 domains. Furthermore, with AcrIIA4 and AcrIIA2 loading, the HNH and RuvC domains were identified as strongly interconnected communities, and the information flow between these domains was enhanced compared to dsDNA loading. This was evidenced by the connection and division between communities 1, 14, and 15 in AcrIIA4 loading and communities 1, 12, 13, and 14 in AcrIIA2 loading (Table S2). Thus, AcrIIA4 and AcrIIA2 can induce more robust signal transduction between the domains of SpyCas9.

Node betweenness centrality, based on the number of shortest paths that traverse the node, is a topological measure of the functional and structural significance of individual residues in protein structures, enabling the identification of key residues involved in signal transduction and allosteric regulation. Residues Leu52 and Gly56 displayed the highest node betweenness among the four systems (Fig. S11), highlighting their importance as critical nodes for transmitting inhibition information. In addition, the observed high node betweenness of polar but uncharged residues within the REC and RuvC domains in the dsDNA-, AcrIIA4-, and AcrIIA2-binding systems also implies their critical role in facilitating the flow of inhibition information across these domains (Fig. S11).

To elucidate the intricate information transfer pathway between the RuvC and HNH domains that underlies the outward reorientation of the HNH domain induced by AcrIIA4 and AcrIIA2 and to investigate the coordination of the catalytic domains during this process, we computed the connecting pairs of catalytic residues of the RuvC and HNH domains. Specifically, we identified the shortest pathways by considering the catalytic residues of the RuvC domain (Asp10, Glu762, His983, and Asp986) as the “source” and the catalytic residue of the HNH domain (His840) as the “sink.” The obtained signaling pathways are represented on the 3D structure of SpyCas9 using lines, while spheres are used to identify the pathway residues’ Cα atoms (Fig. 8d–f and Table S3).

Upon examining the impact of dsDNA loading, we observed that information propagation was facilitated through the L1 loop (residues 765–780), a pivotal hinge region that connects the HNH and RuvC nuclease domains. This observation is consistent with the established experimental evidence that L1 operates as an allosteric effector, modulating the communication between the two aforementioned domains [32], [48]. Generally, AcrIIA4 and AcrIIA2 binding are both different from dsDNA loading, and information transferred between the four catalytically essential residues (Asp10, Glu762, His983, and Asp986) of RuvC and the catalytically essential residue His840 of the HNH nuclease domain mainly via the RuvC-III domain. Moreover, we found a significant difference in the pathway length (PL) of the “shortest pathway” between AcrIIA4 and AcrIIA2 loading, with AcrIIA4 exhibiting a notably shorter PL, indicating that it is a more potent inhibitor than AcrIIA2 [47].

## 4 Conclusion

Long-timescale MD simulations, spanning over multi-microsecond time scales (>10 μs), have elucidated the inhibitory mechanisms of AcrIIA4 and AcrIIA2 from an atomic-level perspective. These simulations have revealed the conformational plasticity of SpyCas9 upon interaction with the Acr proteins AcrIIA4 and AcrIIA2 and have identified critical dynamic determinants underlying the extensive conformational transitions that transpire during the binding process of these Acr proteins. Upon association with AcrIIA4 and AcrIIA2, the conformational transition of SpyCas9 adopts a “departing” state, which transitions to an “approaching” conformational state upon binding with dsDNA. This complex behavior is underpinned by the tightly coupled and precisely orchestrated movements of the protein domains that work synergistically to elicit the substantial conformational modifications critical for the AcrIIA4 and AcrIIA2 association. The binding of AcrIIA4 and AcrIIA2 induces significant conformational rearrangement of the PI and REC2 domains, leading to the departure of the HNH domain away from the central concave region responsible for RNA/DNA heteroduplex accommodation with varying degrees of magnitude. This ultimately results in a decrease in the catalytic activity of SpyCas9. However, due to the limited simulation time scale, the fully inhibited structures of SpyCas9-sgRNA-AcrIIA4 and SpyCas9-sgRNA-AcrIIA2 were not completely captured. This presents a challenge for future investigations to conduct continuous large-scale MD simulations of complex macromolecular systems, such as CRISPR-Cas9.

Our findings also revealed that the SpyCas9-sgRNA complex displays the weakest inter-domain correlation compared to the REC and NUC lobes, which exhibit a more robust correlation upon binding to the substrate DNA, AcrIIA4, and AcrIIA2. This result highlights the crucial role of the REC lobe in coordinating the nuclease activity in the context of AcrIIA4- and AcrIIA2-induced inhibition mechanisms. Moreover, the enhanced correlation between the HNH and REC domains suggests “cross-talk” activity in the AcrIIA4- and AcrIIA2-binding systems. Notably, the collective and coordinated movements of the individual SpyCas9 domains, as indicated by their coupled motions and essential dynamics, allow for concerted motions in various directions necessary to accommodate the conformational transitions required for binding substrate DNA or Acr proteins. Furthermore, residue contact, cross-correlation, hydrogen analyses, and per-residue binding energy decomposition analysis were employed to identify key residues involved in the binding of AcrIIA4 and AcrIIA2 to sgRNA-bound SpyCas9. Our results demonstrate the crucial role of Arg1333 and Arg1335 in PAM recognition, which are highly targeted by both AcrIIA2 and AcrIIA4, as evidenced by high cross-correlation coefficients and extensive hydrogen bonding. These findings corroborate those of previous crystallographic studies and highlight the significance of these residues in the binding process [25], [26], [28]. Additionally, we found previously overlooked critical residues in the SpyCas9 interaction with AcrIIA4 (Asn14, Asp54, Thr770, Gln771) in the crystal structure. Similarly, in the interaction with AcrIIA2, key residues (Asn14, Asp54, Ser55, Lys775, Arg1212, Glu1219) were uncovered. This comprehensive analysis enhances our understanding of the intricate molecular interactions between SpyCas9 and both AcrIIA4 and AcrIIA2, offering new ideas for SpyCas9 modification in biological experiments.

Finally, our investigation revealed the intricacies of the interaction network that governs the binding of AcrIIA4 and AcrIIA2 to SpyCas9 and the subsequent propagation of inhibitory signals to the SpyCas9 endonuclease. The extensive complementarity between these molecules establishes a highly coordinated network wherein the correlated motion of functional domains is critical in transducing inhibitory signals. The relocation of the HNH domain in the presence of the inhibitor is likely a complex process involving multiple structural and dynamic factors. Through the analysis of residue-level communication pathways coordinating functional synchrony and inhibitory signaling, we found that the L1 loop plays a crucial role in transmitting information between the HNH and RuvC domains upon dsDNA binding, whereas the signal is transmitted through RuvC-III upon AcrIIA4 and AcrIIA2 bindings. Moreover, the “shortest pathway” between AcrIIA4 and AcrIIA2 loading exhibited a significant difference in PL, with AcrIIA4 exhibiting a notably shorter PL, indicating AcrIIA4 is a superior inhibitor compared to AcrIIA2 [47].

In conclusion, our study provides a comprehensive and dynamic perspective on the intricate inhibition mechanism of SpyCas9 by the anti-CRISPR proteins AcrIIA4 and AcrIIA2. These findings have far-reaching implications and could potentially drive the development of a novel therapeutic strategy and genome editing for targeting the CRISPR-Cas system, offering a promising avenue for future research and innovation in this field.

## CRediT authorship contribution statement

**Shuixiu Wen:** Data curation, Visualization, Investigation, Writing-Original draft preparation, Writing-Reviewing and Editing. **Yuxin Zhao:** Validation, Investigation, Writing-Reviewing and Editing. **Xinyu Qi:** Validation, Writing- Reviewing and Editing. **Mingzhu Cai:** Writing- Reviewing and Editing. **Kaisheng Huang:** Methodology, Writing- Reviewing and Editing. **Hui Liu:** Conceptualization, Methodology, Writing- Reviewing and Editing, Supervision. **De-Xin Kong:** Conceptualization, Writing- Reviewing and Editing, Supervision, Funding acquisition.

## Declaration of Competing Interest

The authors declare that the research was conducted in the absence of any relationships that could be construed as potential conflict of interest.
