# Supplementary material for: Conformational plasticity of SpyCas9 induced by AcrIIA4 and AcrIIA2: Insights from molecular dynamics simulation
Source: Comput Struct Biotechnol J. 2023 Dec 27;23:537–48. doi: 10.1016/j.csbj.2023.12.030 (PMC10791570; doi:10.1016/j.csbj.2023.12.030)
Supplement: Supplementary file 1 — Supplementary material [file mmc1.docx]

Supplementary Information

Conformational Plasticity of SpyCas9 Induced by AcrIIA4 and AcrIIA2: Insights from Molecular Dynamics Simulation

Shuixiu Wen, Yuxin Zhao, Xinyu Qi, Mingzhu Cai, Kaisheng Huang, Hui Liu*,

De-Xin Kong*

National Key Laboratory of Agricultural Microbiology, Agricultural Bioinformatics Key Laboratory of Hubei Province, College of Informatics, Huazhong Agricultural University, Wuhan, P. R. China.

***Corresponding authors:**

**Hui Liu**

Email: huiliu@mail.hzau.edu.cn;

**De-Xin Kong**

Email: dxkong@mail.hzau.edu.cn.


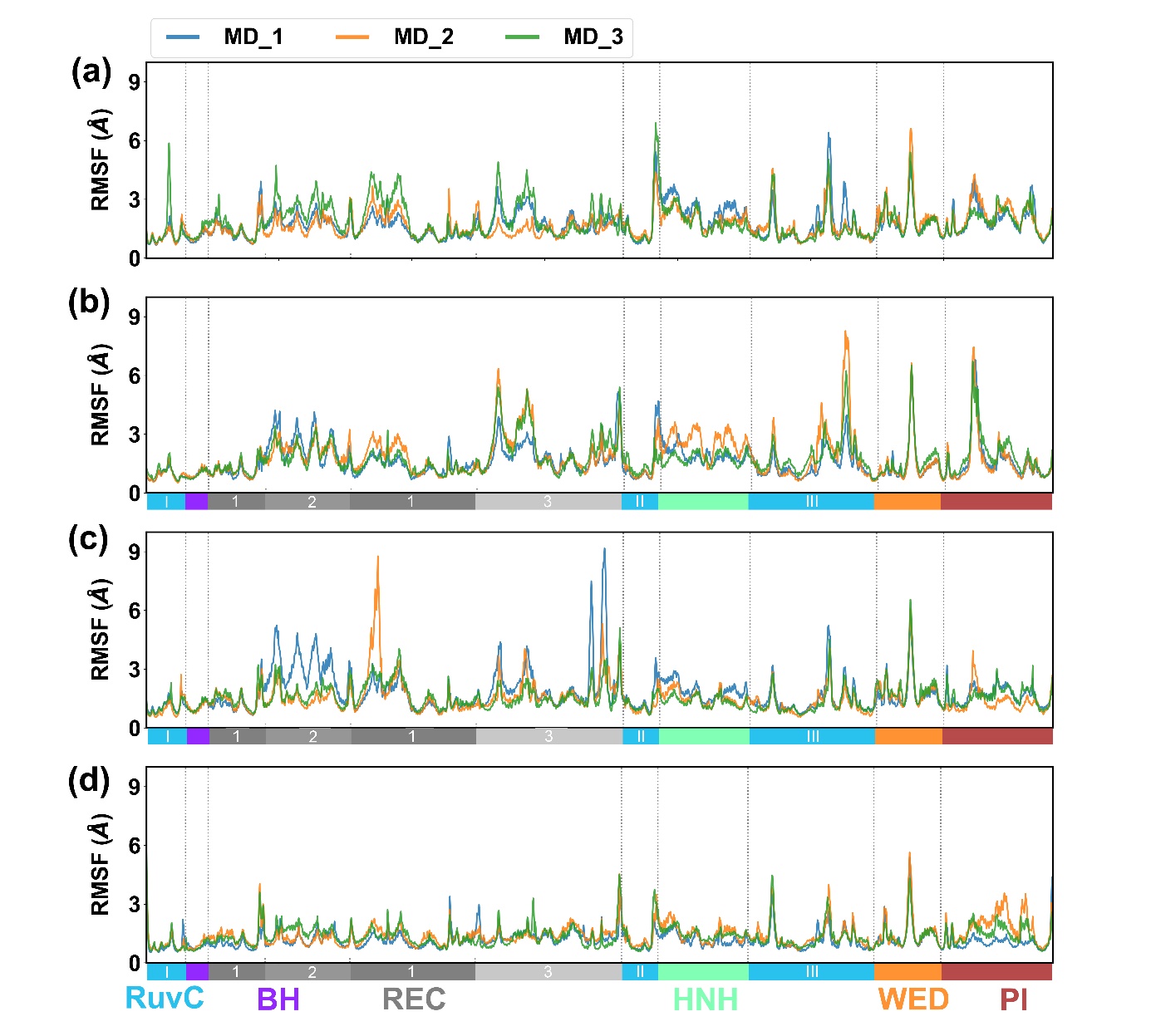


**Figure S1.** Root Mean Square Fluctuations (RMSF) profiles calculated considering the SpyCas9 Cα atoms of SpyCas9-sgRNA (a), SpyCas9-sgRNA-dsDNA (b), SpyCas9-sgRNA-AcrIIA4 (c), SpyCas9-sgRNA-AcrIIA2 (d). The protein sequence is depicted at the bottom of the graphs, with distinct protein domains highlighted using different colors.


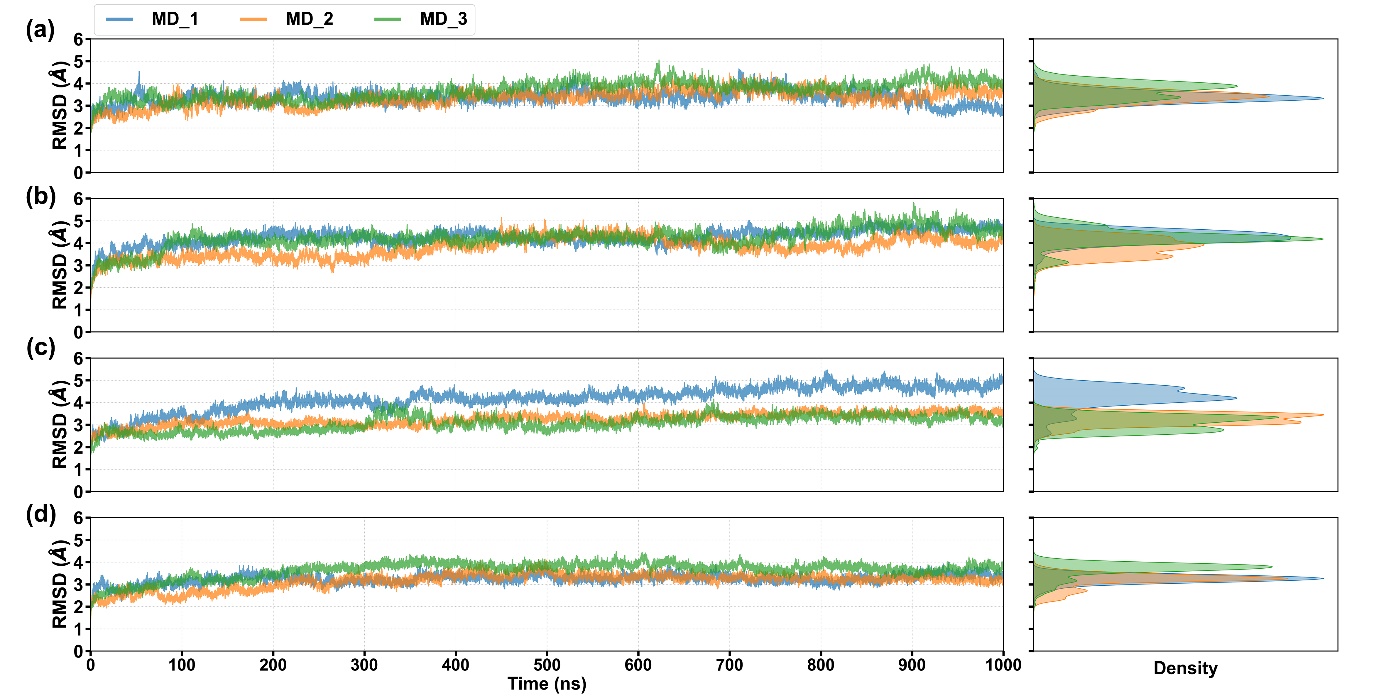


**Figure S2.** Root Mean Square Deviation (RMSD) profiles calculated considering the SpyCas9 Cα atoms of SpyCas9-sgRNA (a), SpyCas9-sgRNA-dsDNA (b), SpyCas9-sgRNA-AcrIIA4 (c), SpyCas9-sgRNA-AcrIIA2 (d).


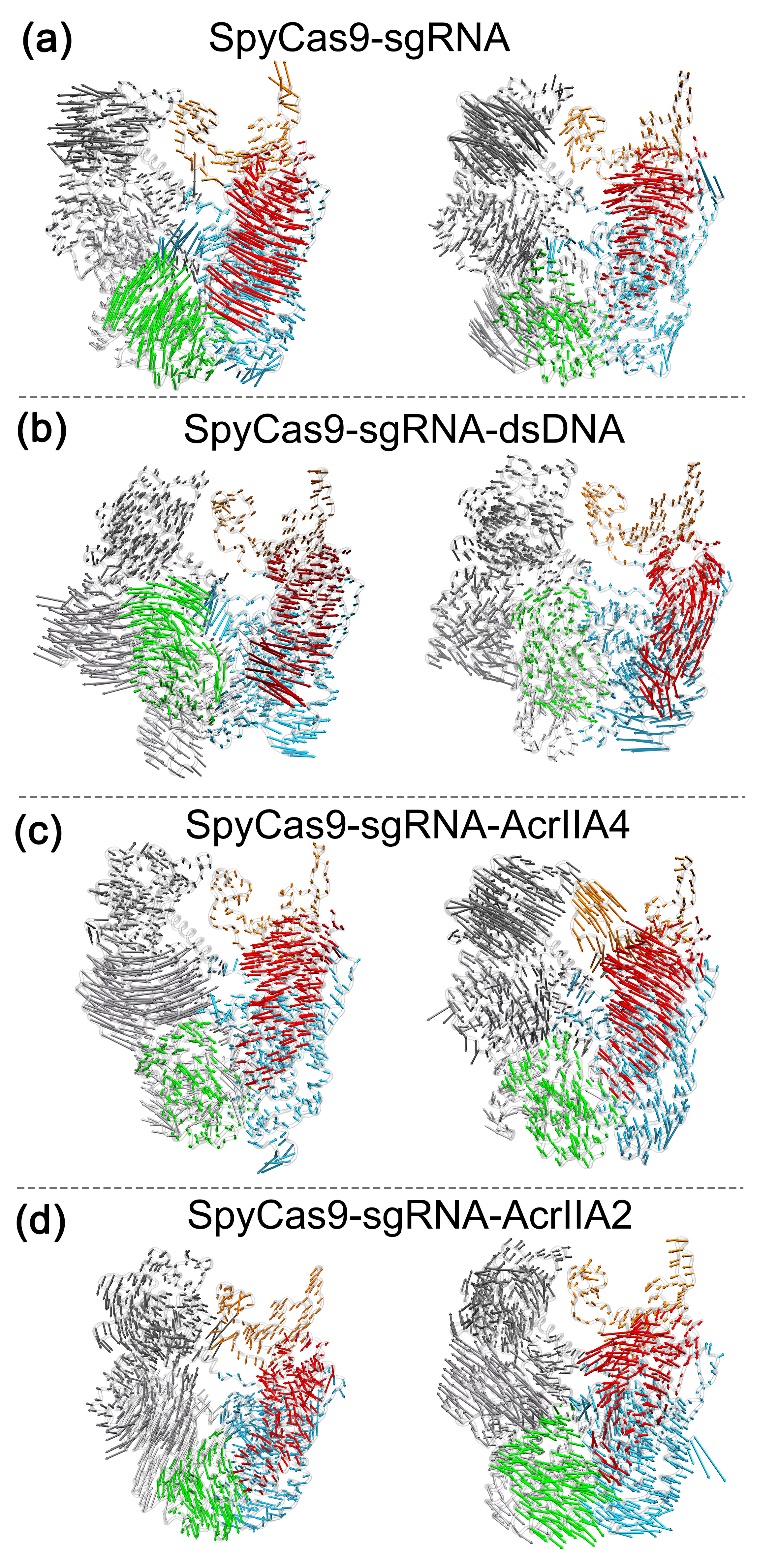


**Figure S3.** Essential dynamics calculated over MD replicas, for the individual protein domains within the following systems: SpyCas9-sgRNA (a), SpyCas9-sgRNA-dsDNA (b), SpyCas9-sgRNA-AcrIIA4 (c), and SpyCas9-sgRNA-AcrIIA4 (d). The essential dynamics were evaluated along PC1, and the resulting motions were visually represented by arrows, indicating both the magnitude and direction of the observed movements.


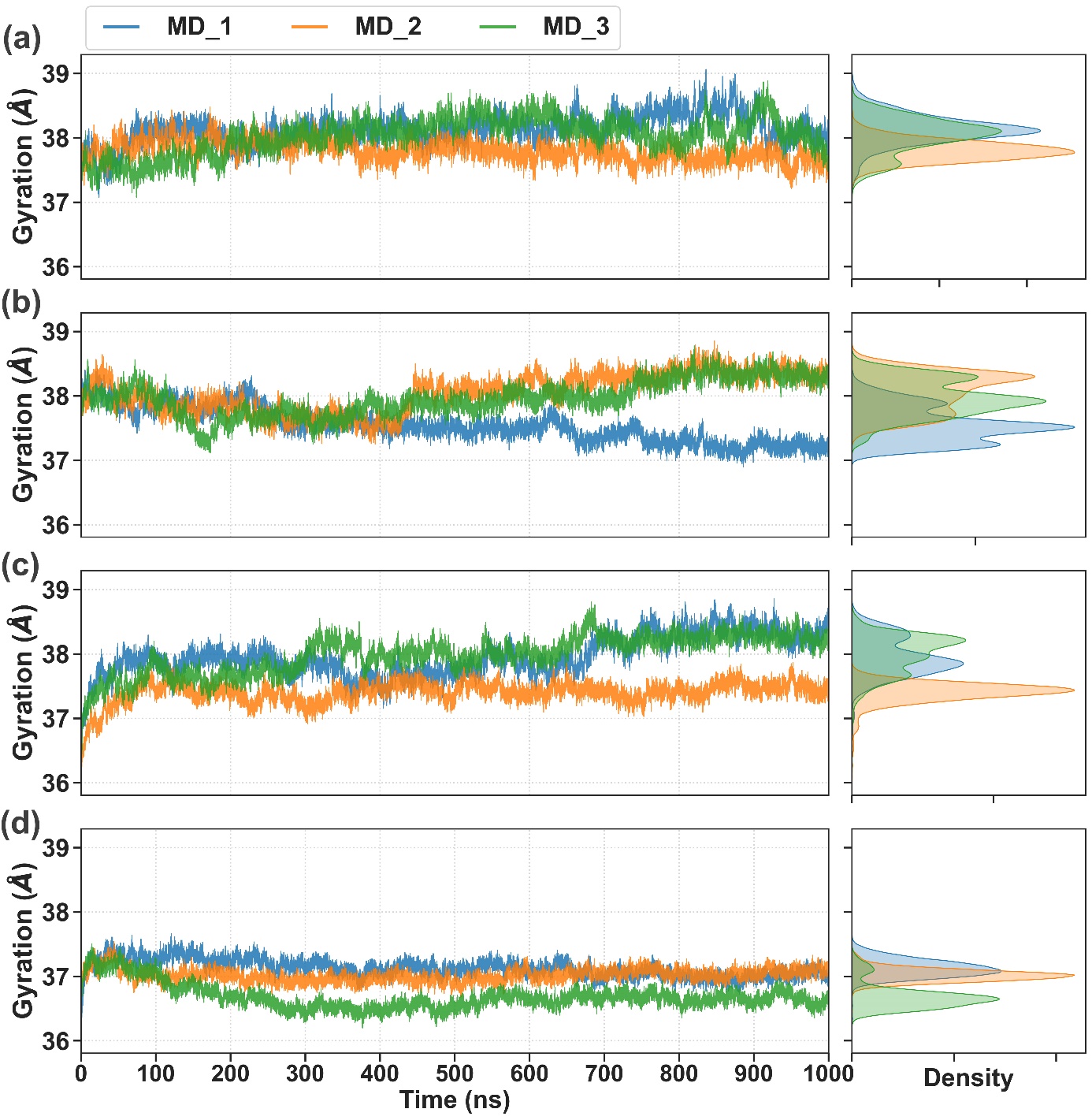


**Figure S4****.** Radius of gyration calculated considering the SpyCas9 Cα atoms of SpyCas9-sgRNA (a), SpyCas9-sgRNA-dsDNA (b), SpyCas9-sgRNA-AcrIIA4 (c), SpyCas9-sgRNA-AcrIIA2 (d).


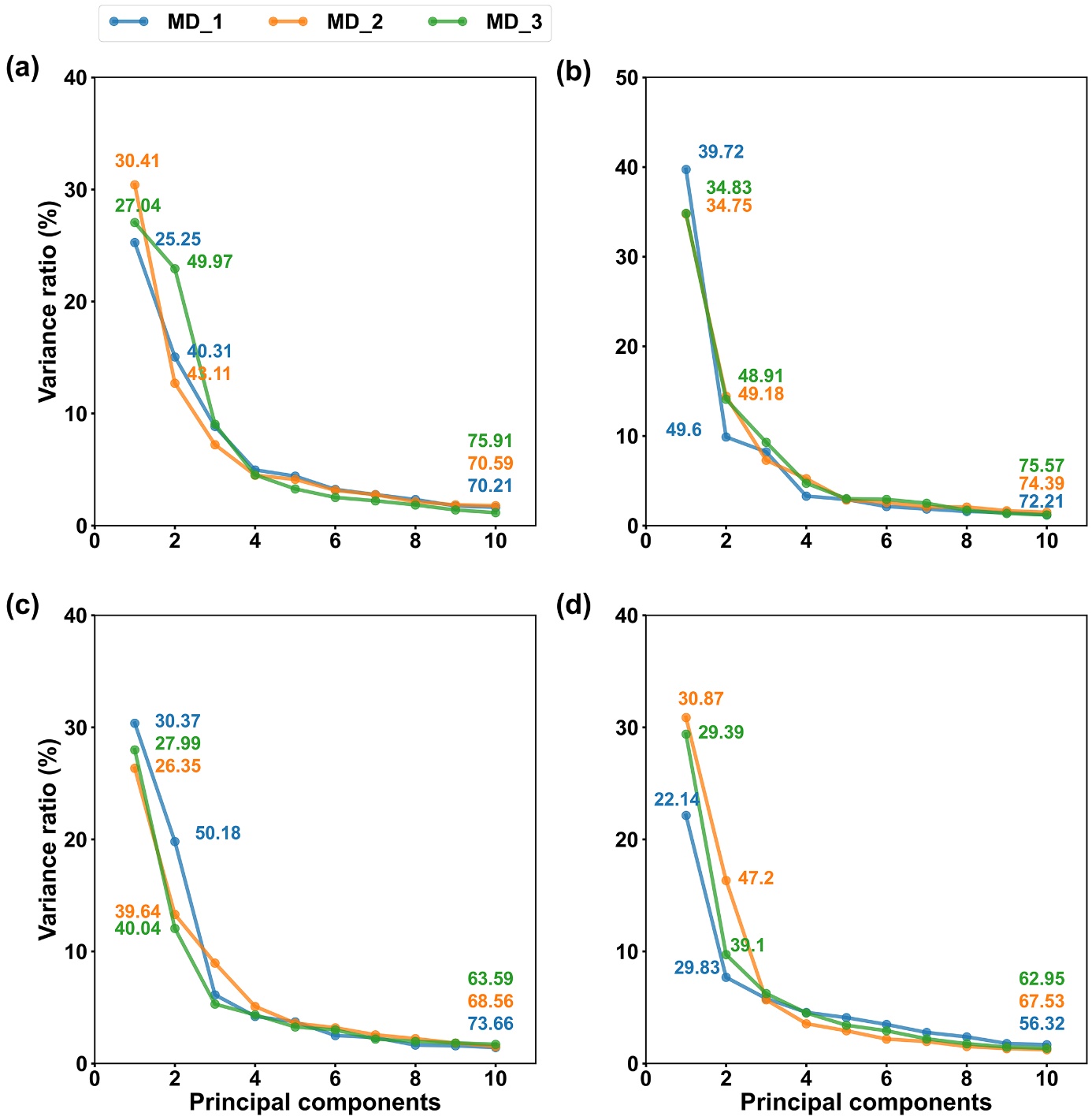


**Figure S5.** Detailed cumulative contribution of the first ten PCs of SpyCas9-sgRNA (a), SpyCas9-sgRNA-dsDNA (b), SpyCas9-sgRNA-AcrIIA4 (c), SpyCas9-sgRNA-AcrIIA2 (d).


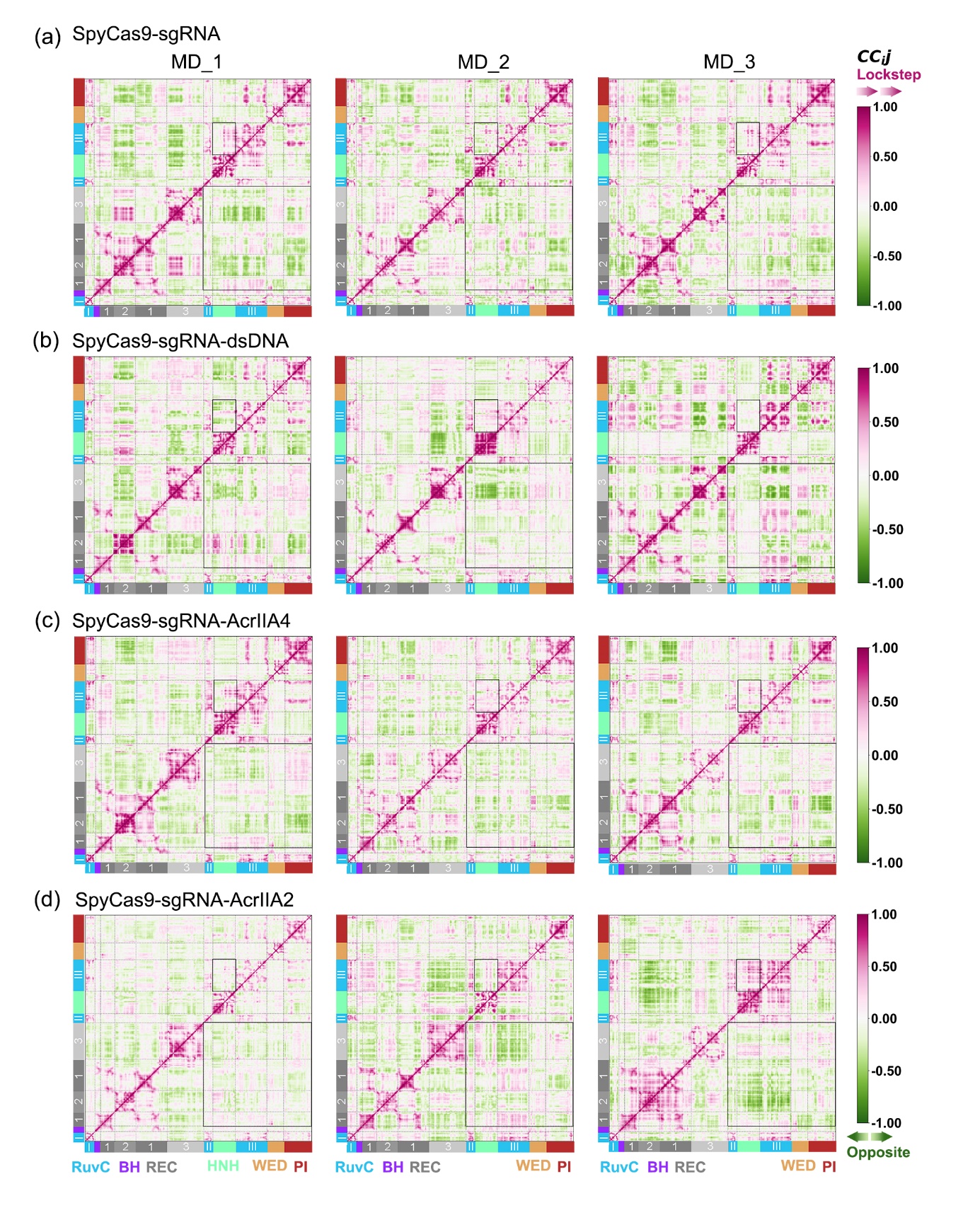


**Figure S6.** Correlated motions of SpyCas9-sgRNA (a), SpyCas9-sgRNA-dsDNA(b), SpyCas9-sgRNA-AcrIIA4 (c), and SpyCas9-sgRNA-AcrIIA2 (d) systems. The magnitude of the CC_ij_ is colored magenta (for CC_ij_ ≥ 0, lockstep motions) to green (for CC_ij_ ≤ 0, Opposite motions). Black boxes are used to highlight anticorrelated CC_ij_ motions between the REC and NUC lobes.


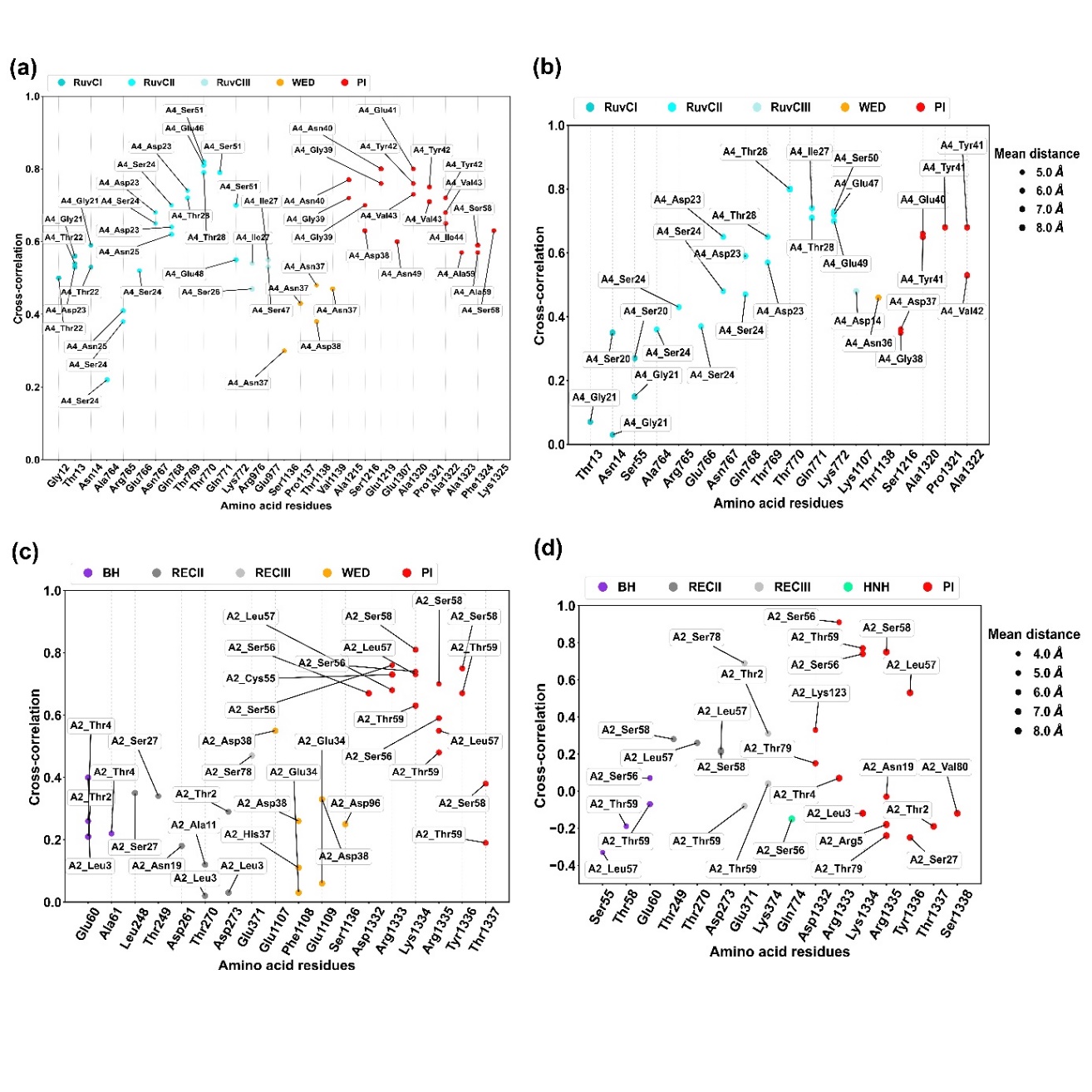


**Figure S7.** Cross-correlation coefficients for contacts frequently between anti-CRISPR (AcrIIA4 and AcrIIA2) and amino acid residues in SpyCas9 protein, calculated over MD replicas. a and b are replicas of SpyCas9-sgRNA-AcrIIA4, c and d are replicas of SpyCas9-sgRNA-AcrIIA2.


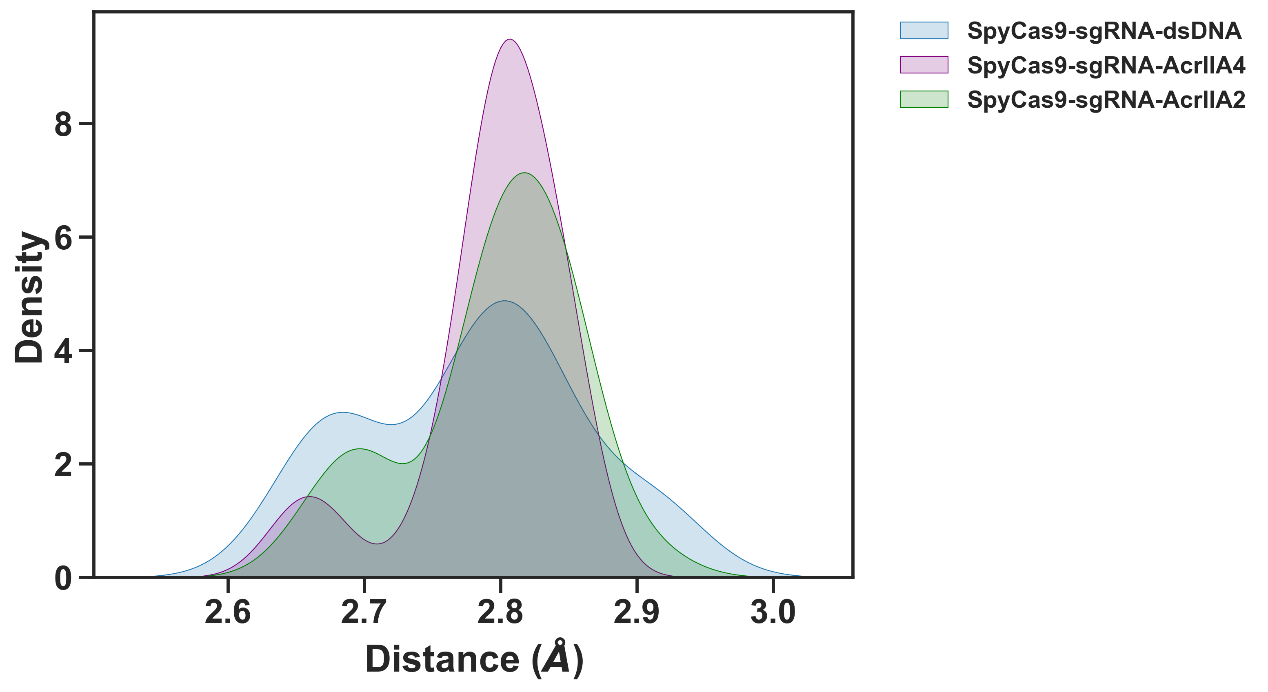


**Figure S8.** Probability distribution of hydrogen bonds.


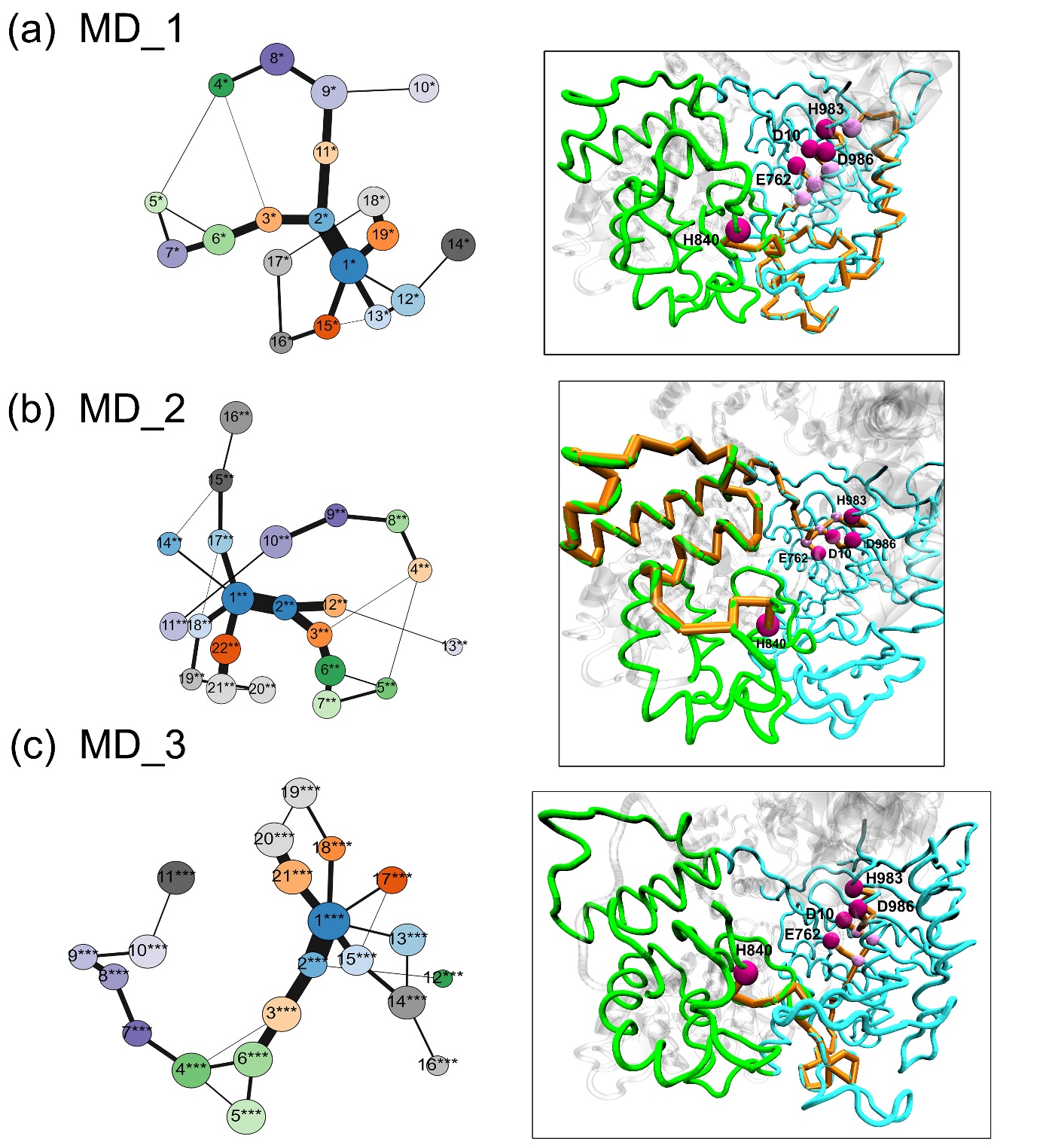


**Figure S9.** Community network representation and “Shortest pathways” linking catalytic residues in the RuvC and HNH domains of SpyCas9-sgRNA replicas. The acquired community networks were represented as interconnected spheres linked by sticks, where the thickness was proportional to the number of shortest paths traversing those junctions.


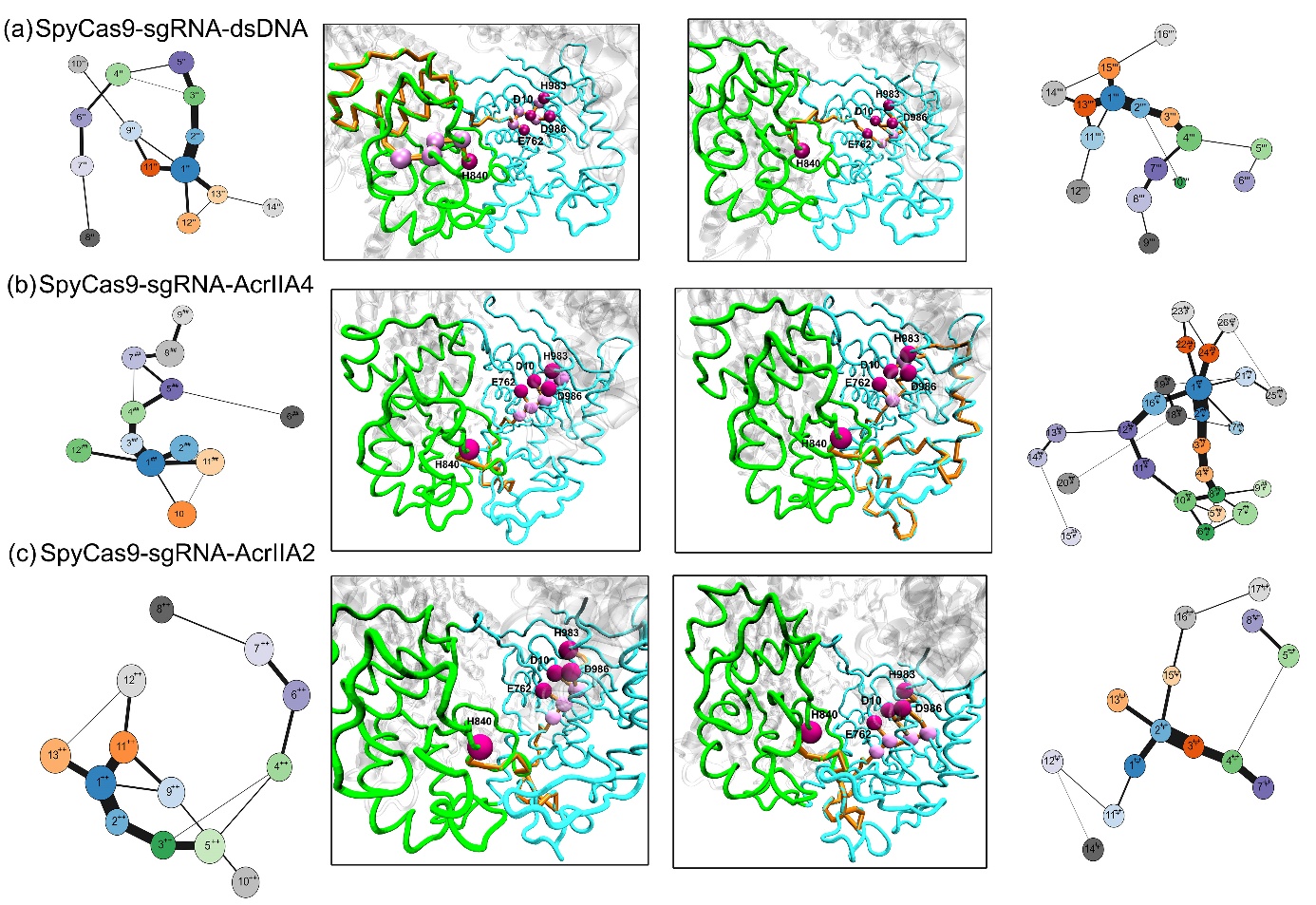


F**igure S10.** Community network representation and “Shortest pathways” linking catalytic residues in the RuvC and HNH domains of SpyCas9-sgRNA-dsDNA(a), SpyCas9-sgRNA-AcrIIA4 (b), and SpyCas9-sgRNA-AcrIIA2 (c) systems, calculated over MD replicas. The acquired community networks were represented as interconnected spheres linked by sticks, where the thickness was proportional to the number of shortest paths traversing those junctions. The characters “ **' ”, “#” , “+”** are used to distinguish community compositions in different systems.


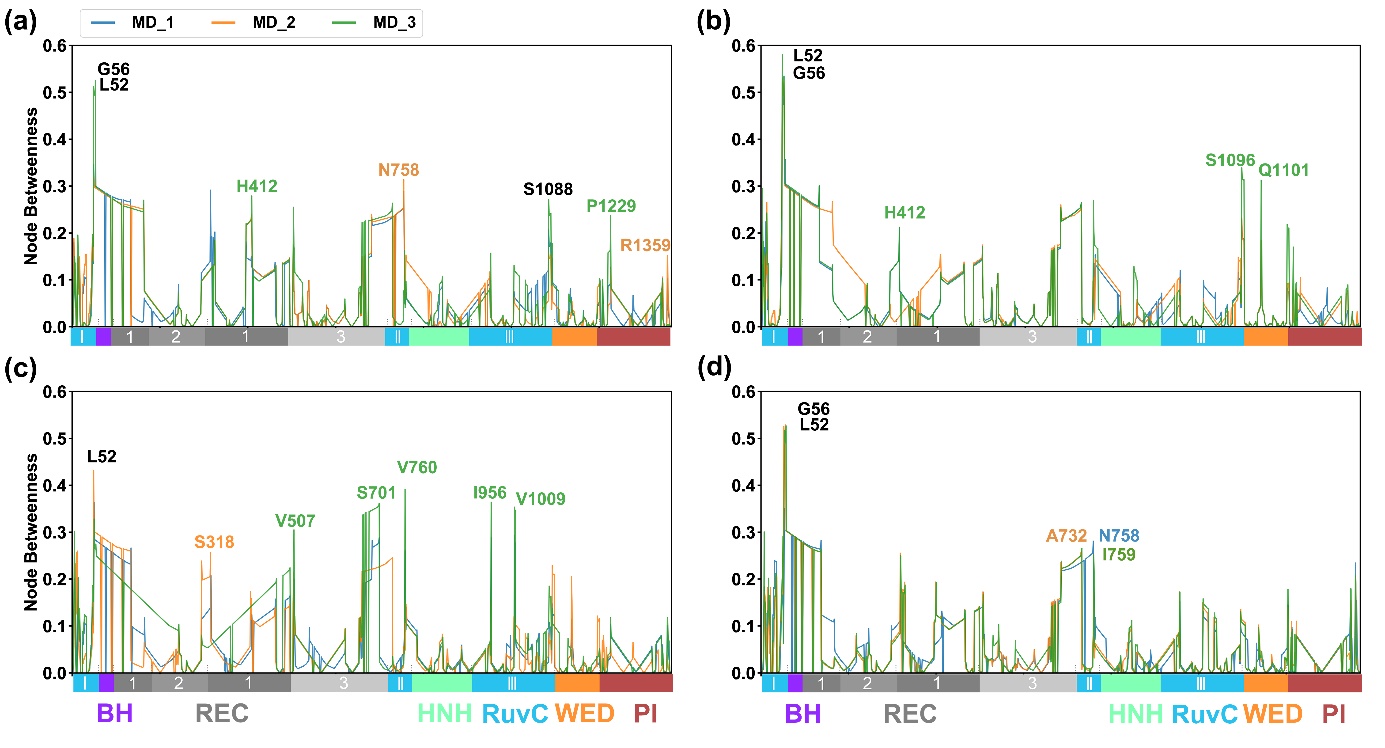


**Figure S11.** Per-residue node betweenness calculated for SpyCas9-sgRNA (a), SpyCas9-sgRNA-dsDNA(b), SpyCas9-sgRNA-AcrIIA4 (c), and SpyCas9-sgRNA-AcrIIA2 (d) systems. The black mark represents the occurrence of the same result in at least two repetitions.


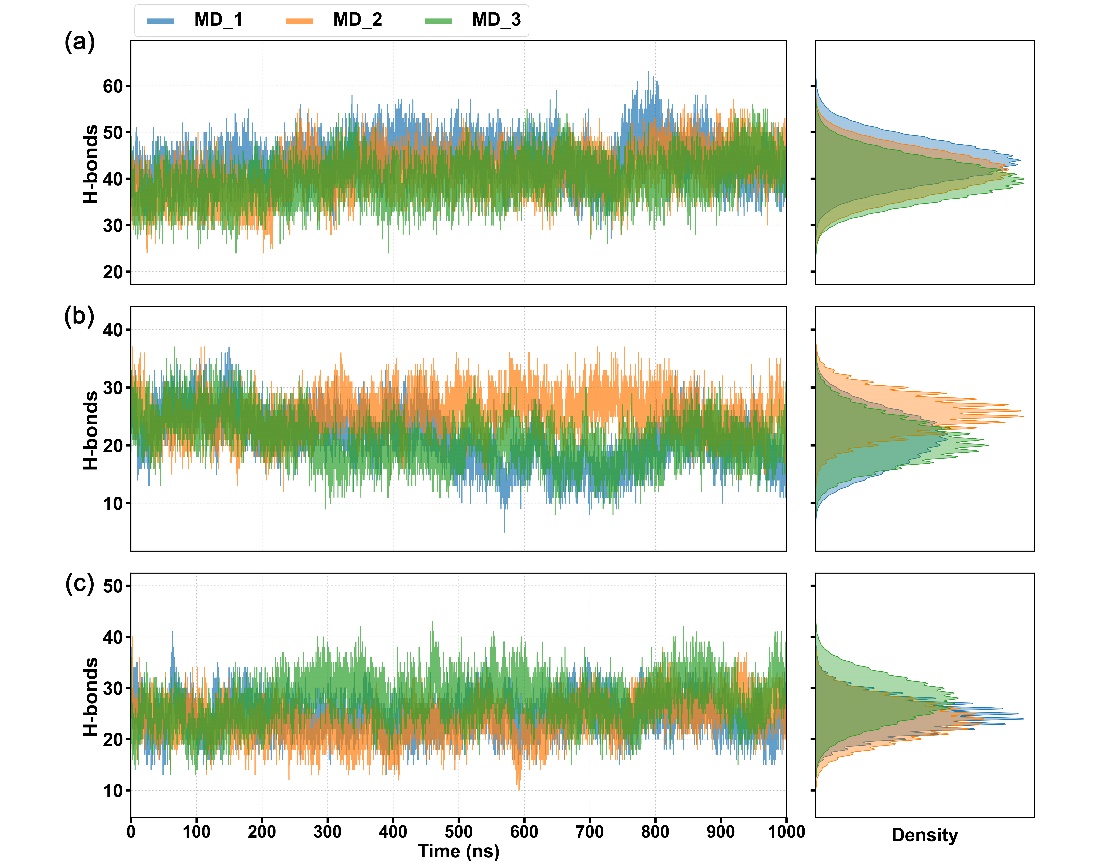


**Figure S12.** Time evolution of the number of hydrogen bonds between dsDNA (a), AcrIIA4 (b), AcrIIA2 (c) and SpyCas9.


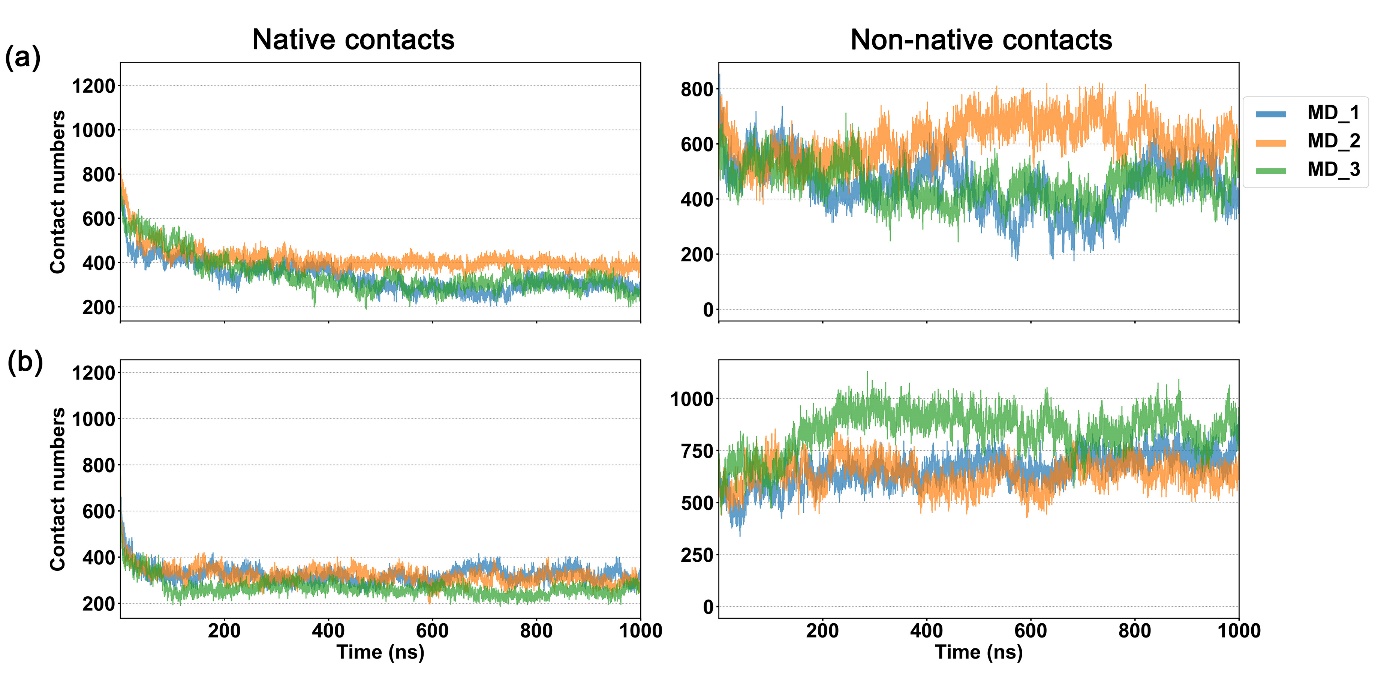


**Figure S13.** Time-dependent evolution of native contacts (detected using the first frame as reference, left column) and non-native contacts (newly formed contacts not detected in the first frame, right column) between SpyCas9 and AcrIIA4 (a), AcrIIA2 (b).


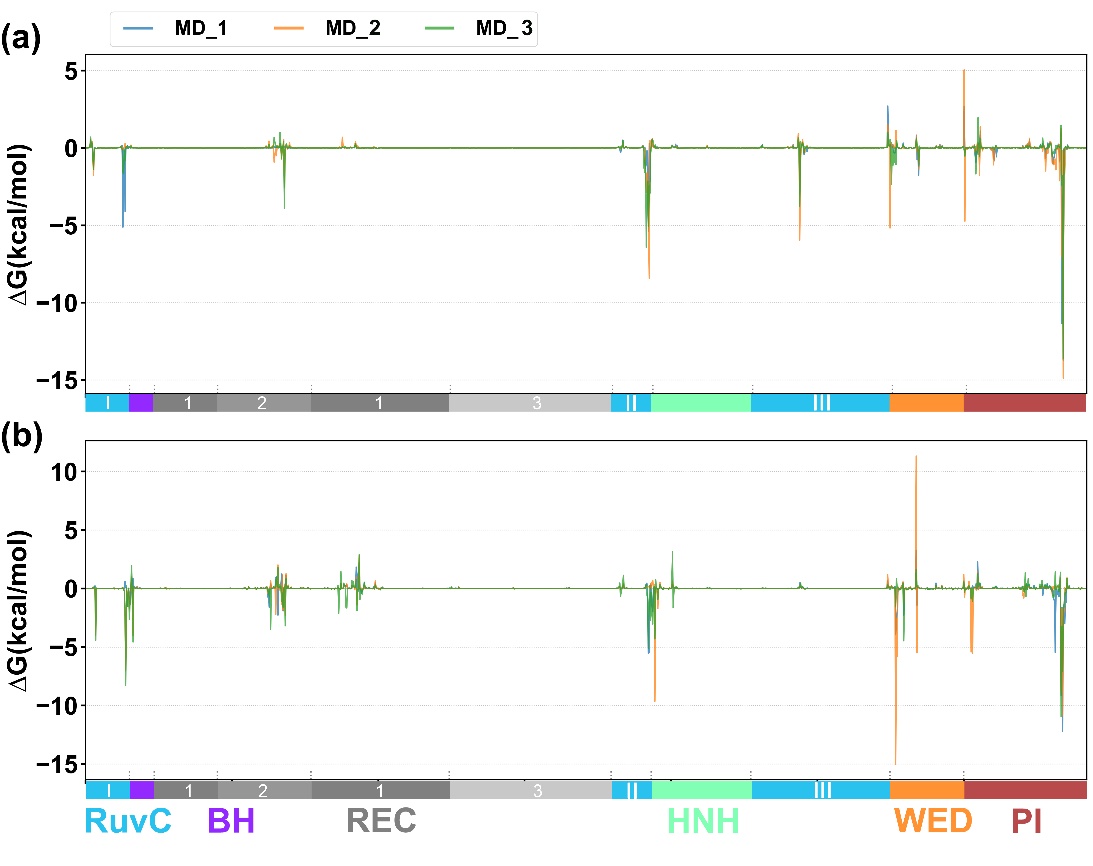


**Figure S14.** Per-residue energy decomposition of SpyCas9 for binding to AcrIIA4 (a) and AcrIIA2(b).

**Table S1.** The composition of residues or nucleotides for the 4 models.

| Systems | Length of protein/Nucleic acid | | | Missing residues of SpyCas9 |
| --- | --- | --- | --- | --- |
|  | SpyCas9 | sgRNA | dsDNA/Acrs |  |
| SpyCas9-sgRNA | 1369 | 85 | - | S0-D2, G1367-D1368 |
| SpyCas9-sgRNA-dsDNA | 1372 | 83 | 40 | G-3-K3, G385, A711-D718, E766-K775, Y1013-I1029, T1051-R1058, Y1242-N1252, L1365-D1368 |
| SpyCas9-sgRNA-AcrIIA4 | 1368 | 98 | 87 | M1-D2, G231, N309-G311, V530-F539, S577-R586, G674, Q695-K705, Q712-Q716, T770-K772, G1367-D1368 |
| SpyCas9-sgRNA-AcrIIA2 | 1369 | 99 | 123 | G715-G717, Q771-G773, G1030-A1032 |

**Table S2.** The composition of community domains in each simulation system.

| Community | SpyCas9-sgRNA | SpyCas9-sgRNA-dsDNA | SpyCas9-sgRNA-AcrIIA4 | SpyCas9-sgRNA-AcrIIA2 |
| --- | --- | --- | --- | --- |
| 1 | RuvC | RuvCI+ RuvCII | RuvC | RuvC |
| 2 | BH | BH+RECIII+RuvCII | RuvCI+PI | RuvCI+PI |
| 3 | RECI | BH+RECI | BH | RuvCIII |
| 4 | RECI | RECI | RECI | BH+RECI |
| 5 | RECII | RECI+ RECII | RECI | RECI |
| 6 | RECII | RECII | RECII | RECI+ RECII |
| 7 | RECI | RECI | RECII | RECII |
| 8 | RECI | RECIII | RECI | RECI |
| 9 | RECIII | RECIII | RECI | RECIII |
| 10 | RECIII | RuvCII | RECIII | RECIII |
| 11 | RECIII + RuvCII | RuvCII+ HNH | RECIII | RECIII+ RuvCII |
| 12 | HNH | HNH | RuvCIII | RuvCII+ HNH |
| 13 | HNH+RuvCIII | RuvCIII | RuvCII | HNH |
| 14 | HNH+RuvCIII | RuvCIII | HNH | HNH+RuvCIII |
| 15 | RuvCIII | WED | HNH+RuvCIII | HNH+RuvCIII |
| 16 | WED | PI | WED | WED |
| 17 | WED | PI | PI | WED |
| 18 | PI | - | - | PI |
| 19 | PI | - | - | - |

**Table S3.** Elucidation of the residue signaling pathway connecting the catalytic sites of HNH (H840) and RuvC (D10, E762, H983, and D986) in SpyCas9.

| Systems | source | sink | PL | Source (RuvC) → Sink (HNH) |
| --- | --- | --- | --- | --- |
| SpyCas9-sgRNA-dsDNA | D10 | H840 | 5.913 | D10 I11 M763 A764 R765 E766 N767 Q768 T769 T770 Q771 K772 G773 Q774 K775 N776 S777 R778 E779 R780 M781 K782 R783 I784 E785 E786 G787 I788 K789 E790 L791 G792 S793 Q794 I795 L796 K797 E798 H799 P800 V801 E802 N803 T804 Q805 L806 Q807 N808 E809 K810 L811 Y812 L813 L816 Q817 N818 G819 R820 D821 T858 L857 V856 K855 H840 |
|  | E762 | H840 | 5.78 | E762 M763 A764 R765 E766 N767 Q768 T769 T770 Q771 K772 G773 Q774 K775 N776 S777 R778 E779 R780 M781 K782 R783 I784 E785 E786 G787 I788 K789 E790 L791 G792 S793 Q794 I795 L796 K797 E798 H799 P800 V801 E802 N803 T804 Q805 L806 Q807 N808 E809 K810 L811 Y812 L813 L816 Q817 N818 G819 R820 D821 T858 L857 V856 K855 H840 |
|  | H983 | H840 | 6.7 | H983 A984 G17 D10 I11 M763 A764 R765 E766 N767 Q768 T769 T770 Q771 K772 G773 Q774 K775 N776 S777 R778 E779 R780 M781 K782 R783 I784 E785 E786 G787 I788 K789 E790 L791 G792 S793 Q794 I795 L796 K797 E798 H799 P800 V801 E802 N803 T804 Q805 L806 Q807 N808 E809 K810 L811 Y812 L813 L816 Q817 N818 G819 R820 D821 T858 L857 V856 K855 H840 |
|  | D986 | H840 | 6.572 | D986 A987 L9 D10 I11 M763 A764 R765 E766 N767 Q768 T769 T770 Q771 K772 G773 Q774 K775 N776 S777 R778 E779 R780 M781 K782 R783 I784 E785 E786 G787 I788 K789 E790 L791 G792 S793 Q794 I795 L796 K797 E798 H799 P800 V801 E802 N803 T804 Q805 L806 Q807 N808 E809 K810 L811 Y812 L813 L816 Q817 N818 G819 R820 D821 T858 L857 V856 K855 H840 |
| SpyCas9-sgRNA-AcrIIA4 | D10 | H840 | 4.512 | D10 L9 G8 A991 V992 V993 G994 T995 A996 L997 I998 K999 K1000 N1066 T1065 E1064 I1063 L1062 P1061 R1060 K1059 E1049 T1048 K1047 F1046 F1045 N1044 M1043 I1042 N1041 S1040 F846 S845 Q844 P843 V842 I841 H840 |
|  | E762 | H840 | 4.631 | E762 I761 V760 I956 V1009 F1008 E1007 S1006 E1005 L1004 K1003 P1002 Y1001 K1000 N1066 T1065 E1064 I1063 L1062 P1061 R1060 K1059 E1049 T1048 K1047 F1046 F1045 N1044 M1043 I1042 N1041 S1040 F846 S845 Q844 P843 V842 I841 H840 |
|  | H983 | H840 | 4.629 | H983 A984 H985 D986 A987 Y988 L989 V992 V993 G994 T995 A996 L997 I998 K999 K1000 N1066 T1065 E1064 I1063 L1062 P1061 R1060 K1059 E1049 T1048 K1047 F1046 F1045 N1044 M1043 I1042 N1041 S1040 F846 S845 Q844 P843 V842 I841 H840 |
|  | D986 | H840 | 4.282 | D986 A987 Y988 L989 V992 V993 G994 T995 A996 L997 I998 K999 K1000 N1066 T1065 E1064 I1063 L1062 P1061 R1060 K1059 E1049 T1048 K1047 F1046 F1045 N1044 M1043 I1042 N1041 S1040 F846 S845 Q844 P843 V842 I841 H840 |
| SpyCas9-sgRNA-AcrIIA2 | D10 | H840 | 5.429 | D10 L9 G8 V760 I956 V1009 Y1010 G1011 D1012 Y1013 K1014 V1015 Y1016 D1017 V1018 R1019 K1020 M1021 K1035 Y1036 F1037 F1038 Y1039 S1040 F846 S845 Q844 P843 V842 I841 H840 |
|  | E762 | H840 | 5.118 | E762 I761 V760 I956 V1009 Y1010 G1011 D1012 Y1013 K1014 V1015 Y1016 D1017 V1018 R1019 K1020 M1021 K1035 Y1036 F1037 F1038 Y1039 S1040 F846 S845 Q844 P843 V842 I841 H840 |
|  | H983 | H840 | 6.284 | H983 A984 H985 D986 A987 L9 G8 V760 I956 V1009 Y1010 G1011 D1012 Y1013 K1014 V1015 Y1016 D1017 V1018 R1019 K1020 M1021 K1035 Y1036 F1037 F1038 Y1039 S1040 F846 S845 Q844 P843 V842 I841 H840 |
|  | D986 | H840 | 5.806 | D986 A987 L9 G8 V760 I956 V1009 Y1010 G1011 D1012 Y1013 K1014 V1015 Y1016 D1017 V1018 R1019 K1020 M1021 K1035 Y1036 F1037 F1038 Y1039 S1040 F846 S845 Q844 P843 V842 I841 H840 |
